# Behavioral Lifestyles and Survival: A Meta-Analysis

**DOI:** 10.3389/fpsyg.2021.786491

**Published:** 2022-02-04

**Authors:** Rocío Fernández-Ballesteros, Elizabeth Valeriano-Lorenzo, Macarena Sánchez-Izquierdo, Juan Botella

**Affiliations:** ^1^Department of Psychobiology and Health, Autonomous University of Madrid, Madrid, Spain; ^2^Department of Social Psychology and Methodology, Autonomous University of Madrid, Madrid, Spain; ^3^UNINPSI Clinical Psychology Center, Comillas Pontifical University, Madrid, Spain

**Keywords:** meta-analysis, mortality, sleep, healthy diet, sleeping, weight control

## Abstract

The aim of the study is to determine the association between Behavioral Lifestyles (regular physical activity, healthy diet, sleeping, and weight control) and longevity in the elderly. A search strategy was conducted in the PsycInfo, Medline, PubMed, Web of Science (WoS), and Scopus databases. The primary outcome was mortality/survival. Four variables (mean of participant's age at the baseline of the study, follow-up years of the study, gender, and year of publication) were analyzed to evaluate the role of potential moderators. Ninety-three articles, totaling more than 2,800,000 people, were included in the meta-analysis. We found that the lifestyles analyzed predict greater survival. Specifically, doing regular physical activity, engaging in leisure activities, sleeping 7–8 h a day, and staying outside the BMI ranges considered as underweight or obesity are habits that each separately has a greater probability associated with survival after a period of several years.

## Introduction

In general, it is genetic and environmental factors which are held to be the main determinants of survival and longevity (Christensen and Vaupel, 1996; WHO, 2002/2012). Among the external factors (such as cultural, socio-economics, educational, or environmental factors), some authors and international biomedical organizations (such as the WHO) have considered personal lifestyles, confounding them with population and aggregate factors, when they should instead be considered as individual behavioral habits developed across the lifespan, in interaction, of course, with genetic and environmental factors but always depending on promotion, selection, rejection.

In this sense, the research project “*Psycho-behavioral factors: The missing link for a new paradigm to account for active longevity (PsyBel)”* is trying to empirically test whether psycho-behavioral factors, such as behavioral life styles, should be considered as a missing link in a new paradigm to account for healthy and active longevity for the simple reason that se while these behaviors are developed across the lifespan, they can be reinforced and promoted, enhanced, or reduced throughout older age (Fernández-Ballesteros, 2017, 2019; Fernández-Ballesteros and Sánchez-Izquierdo, 2019).

### Some Historical Demographic Data

A brief historical portrait combining *healthy lifestyles or habits* with *life expectancy* (based on precedent mortality as a population predictor at birth of a human generation), and *survival* (continued existence or life) and *longevity* (maximum length of life of a given individual or group) shows that since the middle of the nineteenth century, increasing and non-interrupted growth of life expectancy has continued to the present day (Christensen et al., 2009).

Perhaps the clearest perception of the changes happening across longevity is provided in the survival curves by Roser (2013), plotting survival curves for individuals born at different points in time and using cohort life tables, which seems to be a good test of the Fries' hypothesis (see Fries and Crapo, 1981) regarding the *compression* of morbimortality across the *rectangularization* of survival curves; in other words, while <50% of people born in 1851 lived past their 50th birthday, today more than 95% can expect to live longer than 50 years.

According to Riley (2005), average life expectancy at birth (LE) in the European region in 1850 was 36.3 years; in 2001 it was 76.8 years. Experts assume that this phenomenon was produced by a decline in mortality and its corresponding increase in life expectancy and longevity, not only at birth but (from the middle of the twentieth century) across all ages and was mainly due to bio-environmental factors called intrinsic and extrinsic factors by the WHO (2015, 2019). Bio-demography pioneers such as Christensen and Vaupel (1996) stated that: “*approximately one-quarter of the variation in lifespan in developed countries can be attributed to genetic factors. The influence of both genetic and environmental factors on longevity can potentially be modified by medical treatment, behavioural changes and environmental improvements*.” Nevertheless, an intriguing question, as the WHO (2015, 2019) suggested, is the extent to which psycho-behavioral conditions contribute to survival or longevity.

### Living Longer Through Healthy Aging

This extraordinary demographic change: mortality reduction, increasing survival, longevity, as well as life expectancy at birth and throughout the life cycle, occurred in historical association with socio-economic growth, advances in bio-medical and social development, and health and social care, as well as higher education and the general improvement in living conditions.

The WHO (2015, 2017, p. 10) in its *Ageing and Health Reports* summarized the following demography data *projections*: (1) Between 2015 and 2050, the proportion of the world's population over 60 years will nearly double from 12 to 22%. (2) By 2020, the number of people aged 60 years and older will outnumber children younger than 5 years. (3) In 2050, 80% of older people will be living in low- and middle-income countries. (4) The pace of population aging is much faster than in the past. (5) All countries face major challenges to ensure that their health and social systems are ready to make the most of this demographic shift.

Taking into account an aging of aging (aging plus?) panorama, the WHO not only presents useful demographic projections for planning the near future but speculates about how *healthy aging could be understood and enhanced* considering that despite the fact that it is “being widely used in academic and policy circles, […] there is surprisingly little consensus on what this might comprise or how it might be defined or measured” WHO (2015, 2017, p. 41).

The WHO thus posited a new view of *healthy aging*, arguing that it must be considered in a more comprehensive and global sense (in fact, overcoming a bio-medical traditional perspective), connecting both the *life-course*—as a scenario for the development of the person in interaction with his/her context as well as the most important expression of health—and *functionality* in old age. Thus, *healthy aging* is defined as *the process of developing and maintaining the functional ability that enables well-being in older age* [see Figure 2.1, WHO (2017, 2019, p. 42)]. It is made up of the intrinsic capacity of the individual, relevant environmental characteristics, and the interactions between the individual and these characteristics. The WHO thus comes to define *Intrinsic capacity* as *all the physical and mental capacities of an individual*, which means all the basic behavioral repertoires allowing the individual be independent in a given environment, comprising all the factors in the extrinsic world that form the context of an individual's life. In fact, psycho-behavioral conditions seem to be redefined by the WHO as *intrinsic capacities* (as have been defined through the history of psychology) considering that *these intrinsic capacities are determinants of healthy aging* (in interaction with the individual context) and, therefore, for longevity.

In sum, as already stated, the main hypothesis of the *PsyBel Research Project* is to discover the contribution of those *intrinsic mental and physical capacities* such as bio-physical, emotional, attitudinal, motivational, cognitive, and personality characteristics. Thus, *psycho-behavioral factors are supported by the WHO to be determinants for healthy aging* and causal conditions of all demographic indicators of health at individual and population level, taking into consideration that these human psycho-behavioral conditions determine health through the interactions between biological and environmental conditions. This is a new position that must be tested. Measuring the relative contribution of these intrinsic psycho-behavioral conditions to survival, mortality, and longevity opens a new paradigm and requires that not only bio-medical and environmental factors be taken into consideration but also interactive psycho-behavioral determinant of aging and health. The *PsyBel Project* attempts to explore this relevant area and this meta-analytic study is a small first step forward in this direction. Thus, we start with a set of behavioral lifestyles since they are considered by Hendriks and Hatch (2006) as “the result of personal characteristics: individual attributes, aptitudes, capacities, the set of skills and competencies labeled human capital thought to channel individual choices” (Hendriks and Hatch, 2006, p. 302).

### Demographic Revolution and Healthy Lifestyles Habits

There is no academic definition of *healthy lifestyles* or what *healthy habits formally* means. The WHO (1998) stated that “a healthy lifestyle is a way of living that lowers the risk of being seriously ill or dying early” adding that “scientific studies have identified certain types of behavior that contribute to the development of non-communicable diseases and early death” (WHO, 1998, p. 2). In fact, some of these, such as smoking and drinking alcoholic beverages, are considered risk factors for premature death. Nevertheless, lifestyles to be promoted depend on the target population to be trained. If our panorama is *aging*, our objective is to enlarge healthy and active life to 100 years, preventing disability. Moreover, healthy lifestyles are considered *habits* or in other words sets of attitudes and behaviors learnt early in the course of life with very high reinforcing power to the individual and provoking strong habits of *adherence* (as distinguished from sporadic activity) required for improving health.

It is worth remembering when healthy lifestyles started. Socio-historical and demographic events began to lead to an extraordinary growth of life expectancy in the middle of the nineteenth century. At this time, mortality in women was very high because of death from puerperal fever. Dr. Philipp Semmelweis, a Hungarian gynecologist, was working in a Viennese hospital. He observed that gynecologists went directly from the dissection room to the delivery room without taking any antiseptic measures. He then discovered that the incidence of puerperal fever could be drastically cut by the use of hand disinfection in obstetrical clinics when the obstetric doctor and matron started washing their hands carefully before proceeding to the birth.

A century after those events, the concept of lifestyles or healthy life habits gained widespread importance through the link to the Alameda County Study, which started in 1965 and has continued in several waves until the present day. It was designed to investigate normal daily habits, including social relationships, in order to detect risk factors for poor health and mortality in everyday life. The first wave comprised 6,928 participants who completed questionnaires and were followed at intervals for up to 20 years after the initial investigation.

The study yielded ***seven risk factors***—or health practices—associated with poor physical health and excess mortality: *drinking excessive amounts of alcohol, smoking cigarettes, being obese, sleeping fewer, or more than 7–8 h per night, being physically inactive, eating between meals*, and *not eating breakfast*.

The *Alameda County Study* continued using health questionnaires in 1965, 1973, 1985, 1988, 1994, 1999, and 2005. In their examination of the first collected data, Wingard et al. (1982) found that those who followed healthy habits were shown to be associated with physical health status and mortality in a pioneer longitudinal study initiated in 1965 in Alameda County, CA. These habits are *(1) never smoking; (2) drinking less than five drinks at one sitting; (3) sleeping 7–8 h a night; (4) exercising; (5) maintaining desirable weight for height; (6) avoiding snacks, and (7) eating breakfast regularly*. Although a last systematic review of the Alameda County study findings (Housman and Dorman, 2005) reinforced the importance of these seven healthy behavioral habits, a new set of social relationship behaviors were added, showing a link between those habits and survival and longevity.

Some criticism has also been voiced, as has been emphasized by many authors, Alameda County lifestyles are strongly linked to the social contexts of the USA (see Hankin, 2000). Thus, Blane (1995) underlined several of these seven lifestyles as embedded in the USA life standards, for example: avoiding snacks and eating breakfast regularly. Other authors consider that they are highly influenced by the life course (Schulte and Hser, 2012) starting very early in adolescence and being reduced or even stopping after the age of 60.

The objective of this meta-analysis is to synthesize the results of primary studies that evaluate, through longitudinal designs, the relationship between mortality/survival and four of the main lifestyles considered healthy. These four styles have in common that they are observable through objective measures and that they are modifiable: (1) Regular physical activity; (2) Weight control; (3) Healthy diet; and (4) Sleeping 7–8 h per night. To do this, combined estimates of the effect size are obtained, and their significance is evaluated. In addition, the potential moderating role of four variables related to the study design and the characteristics of the participants is analyzed.

## Methods

This report follows the guidelines of the APA task force recommendations about reporting standards for quantitative research in Psychology, and especially in meta-analysis articles (Appelbaum et al., 2018).

### Search Strategy

A systematic search was performed on five websites that provide access to multiple databases related to academic articles. The websites reviewed were PsycInfo, Medline, PubMed, Web of Science (WoS), and Scopus, up to April 2021. The searching strategy was guided by a specific question: Which healthy lifestyle factors are related to longevity in the elderly? Four healthy lifestyle factors were considered as behavior lifestyles: (1) Regular physical or cognitive exercises; (2) healthy diet; (3) sleeping 7 h; (4) weight control. The selected keywords were: “longevity,” “life expectancy,” “lifestyle,” “healthy lifestyle,” “physical activity,” “exercise,” “diet, healthy,” “body weight,” “sleep hygiene/classification,” “sleep/epidemiology,” “sedentary behavior,” “longitudinal studies,” “follow-up studies,” “prospective studies,” “twin studies,” “meta-analysis,” and “elderly” or “aged.” No time restrictions were imposed. The words selected were introduced as free terms and they were searched in the title, abstract, and keywords boxes. The searching strategy is available in Supplementary Material (Supplementary Table 3).

### Inclusion and Exclusion Criteria

**Figure 1:**
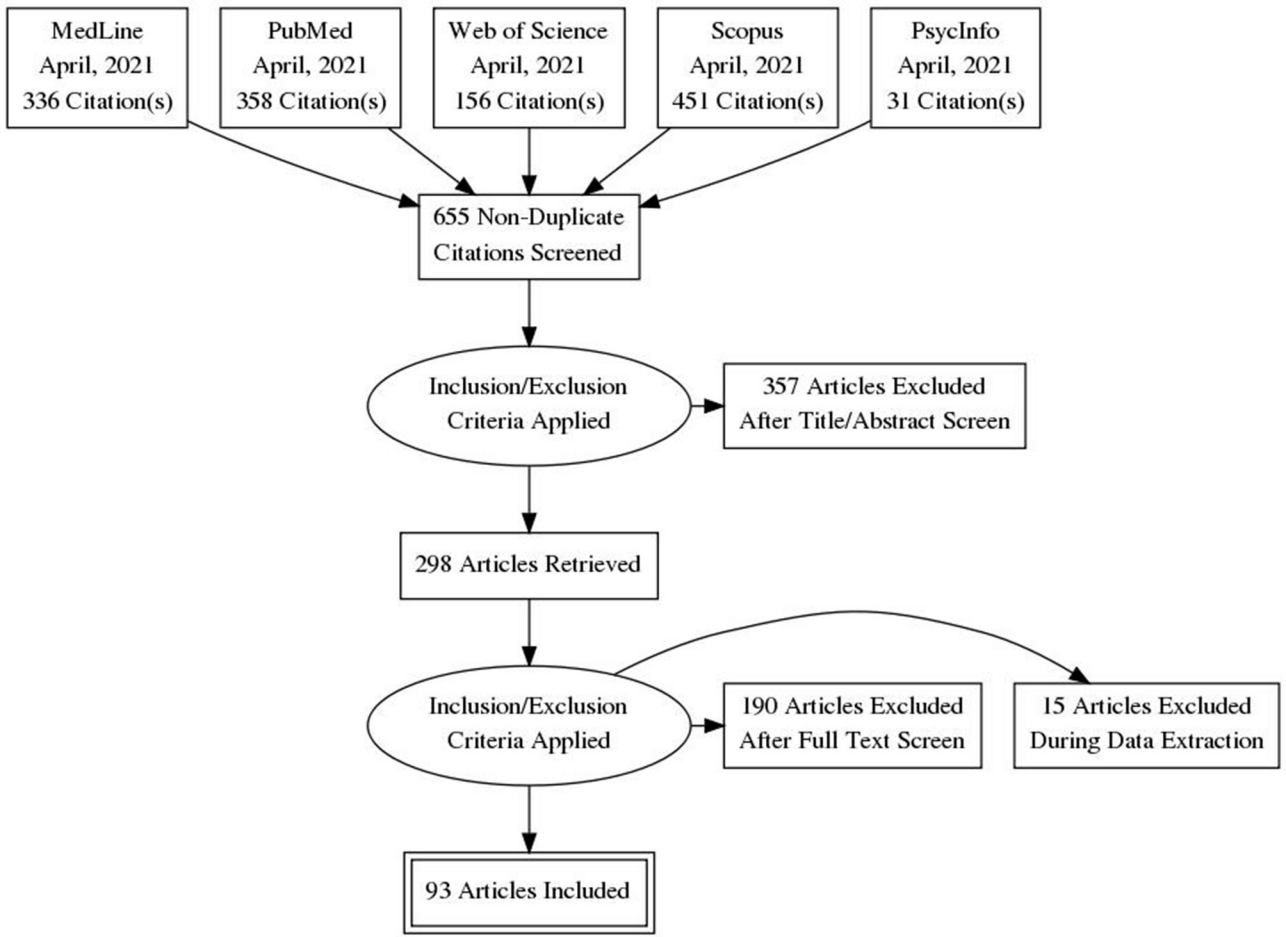
Flow diagram of the study selection.

The inclusion criteria were as follows: (1) the study assessed the association between the selected healthy lifestyle factors on longevity; (2) the target population of the study was focused on the elderly; (3) the study involved outcome indicators as a measure of one or more of the four healthy lifestyle factors; (4) the design of the study was a prospective study; (5) the study reported statistical results on the association between healthy lifestyle factors and mortality or survival. The exclusion criteria were as follows: (1) the study did not address specifically mortality or survival; (2) the study did not address any of our selected healthy lifestyle factors; (3) the design of the study was not longitudinal; (4) the mean age of the sample population was not elderly, over the study period. Additionally, if two studies were based on the same dataset, even partially, the study with more follow-up years was selected. When a study did not report the statistic that reflects the association assessed and their confidence interval, an exact *p*-value was required to estimate the confidence interval. If the confidence interval or exact *p*-value were not reported, the study was excluded. The flow diagram of the study identification and selection is shown in Figure 1.

### Reviewing Procedure and Data Extraction

Database searches were conducted in April 2021. Potentially eligible studies were selected in two steps; the first step was based on the title and abstract screening. Irrelevant references were removed. The second step was based on the full-text reading of potentially relevant studies. The pre-specified eligibility criteria were checked in both phases. For each reference, the following variables were systematically extracted, and they were entered into a summary table: (1) Author, year; (2) number of follow-up years; (3) sample size at the baseline; (4) percentage of female participants; (5) mean age at baseline; (6) overall death rate; (7) death rate of the reference group; (8) Dataset or project name; (9) participants (10) predictor variables; (11) outcome; (12) Assessment/Instrument(s); (13) Index type [Hazard ratio, odds ratio (OR), relative risk (RR)]; (14) Effect size; (15) Confidence interval of the effect size. The collected data is available through the author's mail account.

### Main Variables

Regular physical exercises, healthy diet, sleeping 7 h, and weight control were the healthy lifestyle factors selected due to their significant association, according to previous literature, with a higher probability or a larger longevity. Also, those factors have been widely studied and are supported by several previous studies.

There were several measures reported by the selected studies. Specially, several ways of operationalizing and quantifying the variables of interest were observed. It should be noted that, the categories of the studies were based on those measures.

Altogether, the physical activity has been measured in various ways, by the practice/absence of the physical activity in the daily routine of the participant, by the frequency of the physical activity practiced (times per week), by the intensity of the activity performed (metabolic equivalent of task—METs), by taking walks on a regular basis, or engaging leisure activities. Based on that criterion, we have done a sub-division within the regular physical exercise factor (see Table 1) for better comprehension: ***(1A) Physical activity (Yes-No)***, which involves studies where the participants were classified in two groups “Physically active” vs. “Not physically active.” ***(1B) Physical activity Frequency***, based on the studies where the physical activity is quantified by the number of times per week performed and categories ranging from Never to Regularly or Often were used. ***(1C) Vigorous Physical activity***, which involves the studies that quantify various types of physical activity in METs (metabolic equivalent of task) or in hours per week, also quantiles (quintiles, quartiles, or terciles) were calculated and those were the references to set cut-off points to build categories. ***(1D) Walking***, based on the studies using the frequency per day or week, the distance of walking, or the walking at a brisk pace in minutes per day as the basis for their proposed categories. ***(1E) Leisure activities or Leisure physical*** ***activities***, which involves the studies that ask about engaging in several leisure activities, and the practice of physical activity may or may not be present as a leisure activity. In those studies, the measure was based on the frequency, the number of activities engaged, or the number or hours invested. It was not possible to select just one category for the focus group, as the cut-off points of the categories differ among studies. However, the Sedentary or Physically Inactive group has been taken as the control group in the five dimensions of physical activity. In the opposite, the highest measure/category reported was considered as the focus group. Despite the previous statement, the HR has been calculated in reference to the medium category in two studies (Ford et al., 2008; Schultz-Larsen et al., 2012), in that scenario the effect sizes are more likely to be less than a HR compared to the low category.

**Table 1:** Means values (and range) in the four moderators studied, grouped according to the factors assessed.

	**Main factors**	**Average of mean age [range]**	**Average of % of women [range]**	**Average of follow-up years (FUY) [range]**	**Average of publication year (PUBY) [range]**
**Regular physical activity**	PA (Yes-No)	71.8 [47.5–92.3]	52.4 [0–100]	13.9 [3–42]	2006 [1990–2018]
	PA frequency	65.5 [38.7–85]	56.8 [0–100]	14.9 [2.2–46]	2008 [1994–2018]
	Vigorous PA	63.1 [38.7–85]	51.6 [0–100]	13.1 [5.1–33]	2013 [1997–2020]
	Leisure activity/leisure physical activity	64.9 [46.8–92.7]	46.2 [0–71.6]	13.4 [2.2–29]	2014 [2000–2020]
	Walking	69.7 [46.6–85.9]	52.5 [0–100]	9.1 [2–16]	2006 [1992–2019]
	**Healthy diet**	59.7 [39–92.3]	51.3 [0–100]	13.6 [4–40]	2012 [2003–2020]
	**Sleeping**	76.3 [54.9–92.7]	54.2 [0–100]	10.8 [9–12]	2009 [2003–2020]
**Weight control**	Obesity	64.6 [49.3–91.8]	48 [0–100]	13.8 [4.3–32.5]	2009 [1991–2018]
	Overweight	60.8 [48.2–93.1]	42.5 [0–100]	14.1 [5–40]	2009 [1991–2016]
	Underweight	64.5 [42.5–91.8]	54 [0–100]	12.8 [4.3–27.5]	2008 [1991–2018]

In the case of the Healthy Diet factor, some studies used the adherence of participants to the Mediterranean diet as a measure, other studies reported the frequency or number of daily servings of fruits and vegetables (WHO recommendations), and other studies focus on measuring the amount of calories consumption. In all studies, the control group was determined by the lowest adherence to the Mediterranean Diet (score), the lowest frequency of fruit and vegetable intake or the lowest number of servings; instead of, the focus group was determined as the opposite category to the control group, for example the highest frequency of fruit and vegetable intake.

Respect of the Sleeping 7–8 h factor, two of the three selected studies established 7–8 sleeping h as the control group and one of them used 6 h as control group; and a greater number or lesser number of sleeping hours, than control group, was considered the focus group.

Regarding the Weight control factor, with body mass index (BMI) as a proxy, the categories used refer mostly to the limits established by the World Health Organization, although in other studies the authors considered the division into quantiles or cut-off points based on the median or mean of the complete sample or by gender sample. For our purpose, a sub-division of Weight control was assumed as follows: ***(4A) Obesity*** represented by a BMI ≥ 30, ***(4B) Overweight*** represented by a 25 ≤ BMI < 30, and ***(4C) Underweight*** represented by a BMI < 20 (or BMI < 22 in some studies). The control group for this factor has been defined as the Normo-weight (20 ≤ BMI < 25) and the three BMI categories mentioned before were considered the focus group. The cut-off points of the categories are comprised in most studies.

It must be mentioned that the selected studies reported analyses based on one or more of the four factors of healthy lifestyle studied in this meta-analysis. In the studies that reported separate estimates by gender, they were taken as two different samples in the same study. Sixty-one studies assessed regular physical activity, 14 studies focused on healthy diet, 3 studies assessed sleeping 7–8 h, and 36 studies assessed control weight (see Supplementary Material, Summary of studies). The studies included in Regular physical activity reported different measures of physical exercises, 13 studies were involved in Physical Activity (Yes–No), 10 studies were related to Physical Activity Frequency, 31 studies were involved in Vigorous Physical Activity, 11 studies focused on Walking, and 12 studies were related to Leisure activities.

The assessments done in the studies and their instruments of measure are described in Supplementary Material (see Supplementary Table 2). Healthy lifestyle habits assessment.

### Effect Size and Statistical Model

Lifestyle indices scores were created in some primary studies. Those indices usually combined two or more of the factors that are examined here. As their combination rarely match, it is not appropriate to synthesize these combined indices. We have only worked with the indices referred to the factors separately for those studies.

The effect size index in this meta-analysis is the *Hazard Ratio* (HR) *for mortality*. The value in the numerator refers always to the condition assessed, and that in the denominator the category for control. Thus, values lesser than one reflect smaller mortality (larger survival), whereas values larger than one reflect larger mortality (smaller survival). The results of the primary studies were generally presented directly as HR values, but were sometimes presented as proportions, RRs, or OR. To convert the OR and/or RR values to HR values, the formulas of Zhang and Kai (1998) (see Okun et al., 2013) were used. The variances were obtained through the reported confidence intervals or the exact *p*-values of the significance tests. The values have been previously transformed to their logarithms for the statistical analysis, in order to have a more symmetric distribution. The results reported below have already been back transformed to the original metric, so that they appear as HR values along the paper, but not in the forest plots, where they appear as LogHR.

Variability between studies was evaluated using the *Q* statistic, as a test of heterogeneity, and the *I*^2^-statistic (Huedo-Medina et al., 2006). For the pooled estimate, the values have been weighted by the inverses of their variances. Random effects models have been assumed, instead of fixed effect model. Random effect models are generally preferred because they are more conservative and allow generalizing the conclusions beyond the specific set of studies analyzed (Borenstein et al., 2010). The specific variance was estimated through the restricted maximum likelihood method.

Meta-regression moderator analyzes were performed to assess four potential sources of heterogeneity: participants' mean age at baseline, length of follow-up, gender (percentage of women), and year of publication.

The risk of publication bias, as reflected in the asymmetry of a funnel plot, was assessed by visual inspection of the figure and some statistical tests, as the Egger's test, the rank correlation test, and the Trim and Fill method. We have also calculated the fail-safe numbers (Rosenthal, 1979). The results of those analyses are summarized in **Table 3**. Finally, the analyzes and figures were performed using the R package “metafor” (Viechtbauer, 2010). We have not applied other methods, such as *p*-uniform, because the number of significant studies was generally too small to obtain stable results (Blázquez et al., 2017).

## Results

### Studies Characteristics

Searching databases resulted in 655 unique records. Of these 655 articles, 357 were excluded in the first step, based on the title and the abstract (Figure 1). Of the remaining 298 articles, 190 were excluded in the second step, based on full texts and 15 were excluded during the extraction stage. This process resulted in a total of 93 articles included in the meta-analysis. Supplementary Table 1 (see Supplementary Material) provides an overview of the 93 studies included.

The median year of publication was 2012 (range 1990–2020). The median sample size was 6,382, ranging from 148 to 654,827. The median of the average age was 64.5 years (38.7–93.1 years). Studies were conducted in Europe (36.6%), United States (34.4%), Asia (12.9%), United Kingdom (9.7%), Australia (2.1%), Central America (1.1%), South America (1%), and United States and Europe at the same time (2.1%). The median follow-up time was 12 years, varying between 2 and 46 years (see Supplementary Table 1).

### Description of the Moderators Assessed by Factor

The means and ranges in the four moderators of the included studies are shown in Table 1.

### Regular Physical Activity

**Figure 2:**
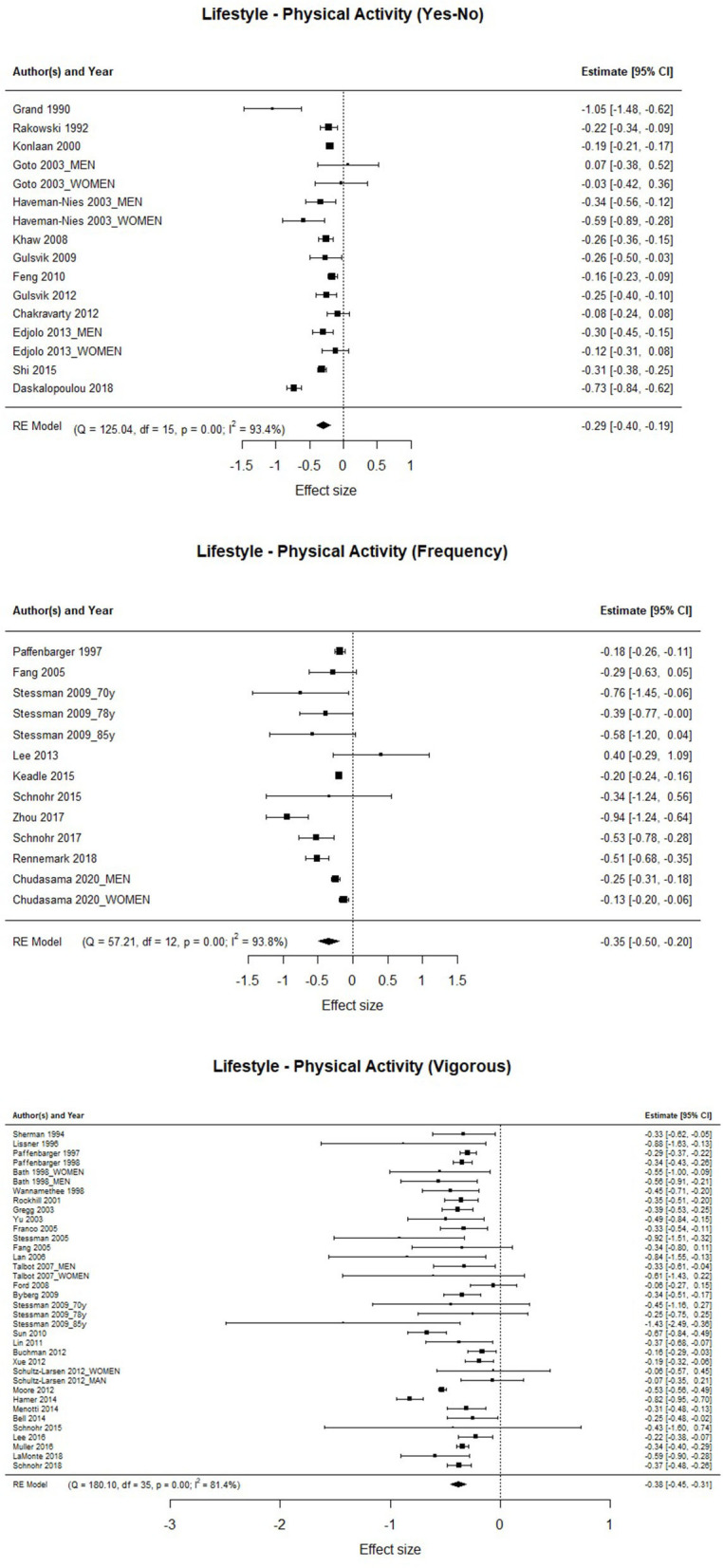
Association between physical activity, frequency of physical activity, and vigorous physical activity, and the risk of mortality.

Figure 2 shows the forest plots with the three groups of studies that provide results on Regular Physical Activity. In the three cases the control group consist of participants who are considered inactive or sedentary. Three focus groups were raised, the first focuses on categorizing physical activity into two opposing groups “If you are physically active” and “Not physically active.” The second focus group worked with categories where the frequency of physical activity was evaluated. And the third focus group considered the physical activity quantified in METs (body energy expenditure) by hours a day or a week. The three focus groups show evidence of a significant association with the risk of mortality [HR_active_= 0.75; 95%CI: 0.672–0.831; HR_frecuency_ = 0.705; 95%CI: 0.609–0.817; HR_vigorous_ = 0.684; 95%CI: 0.639–0.730], supporting the hypothesis of the physical activity as an important factor associated to a significantly smaller rate of mortality. The results also show a significant heterogeneity with the three-focus group [Q_active_ (15) = 125.044, *p* < 0.001; Q_frecuency_ (12) = 57.212, *p* < 0.001; Q_vigorous_ (35) = 180.096, *p* < 0.001].

**Figure 3:**
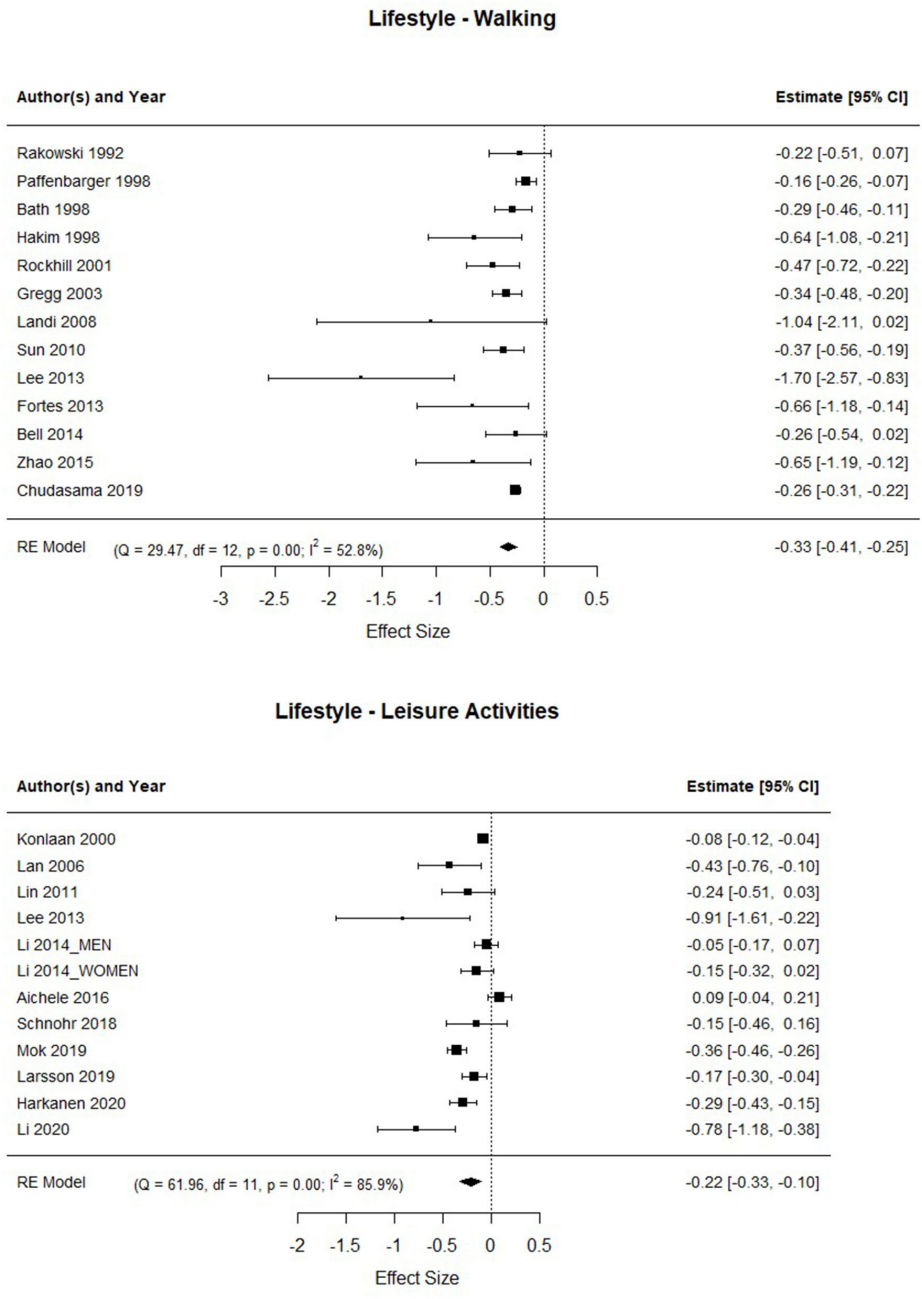
Association between walking regularly, and performing leisure time activities, and the risk of mortality.

The forest plots with the studies involved in Walking and Leisure Activities/Leisure Physical Activities are shown in Figure 3. The Walking control group is conformed with the participants with low activity in regular walks (quantified in <10 min per day or <5 km per week in most studies). There was evidence of a significant association between high walking activity and mortality [HR = 0.72; 95%CI: 0.665–0.783], with the mortality rate being significantly lower, 28% less, than in the group that does not walk regularly.

Regarding the studies referred to the “leisure activities/leisure physical activity,” the control group was integrated by people who rarely perform this type of activity. The results showed a significant association between frequent leisure time activities and/or leisure physical activities and mortality [HR = 0.81; 95%CI: 0.72–0.90], that means participants who frequently engage in leisure time activities and/or leisure physical activities get a mortality risk of 19% lower than people who rarely or never perform those activities. Again, it should be noted that the heterogeneity in both factors is high [Q_Walking_ (12) = 29.471, *p* < 0.001; Q_LeisureActivity_ (11) = 61.964, *p* < 0.001].

### Healthy Diet

**Figure 4:**
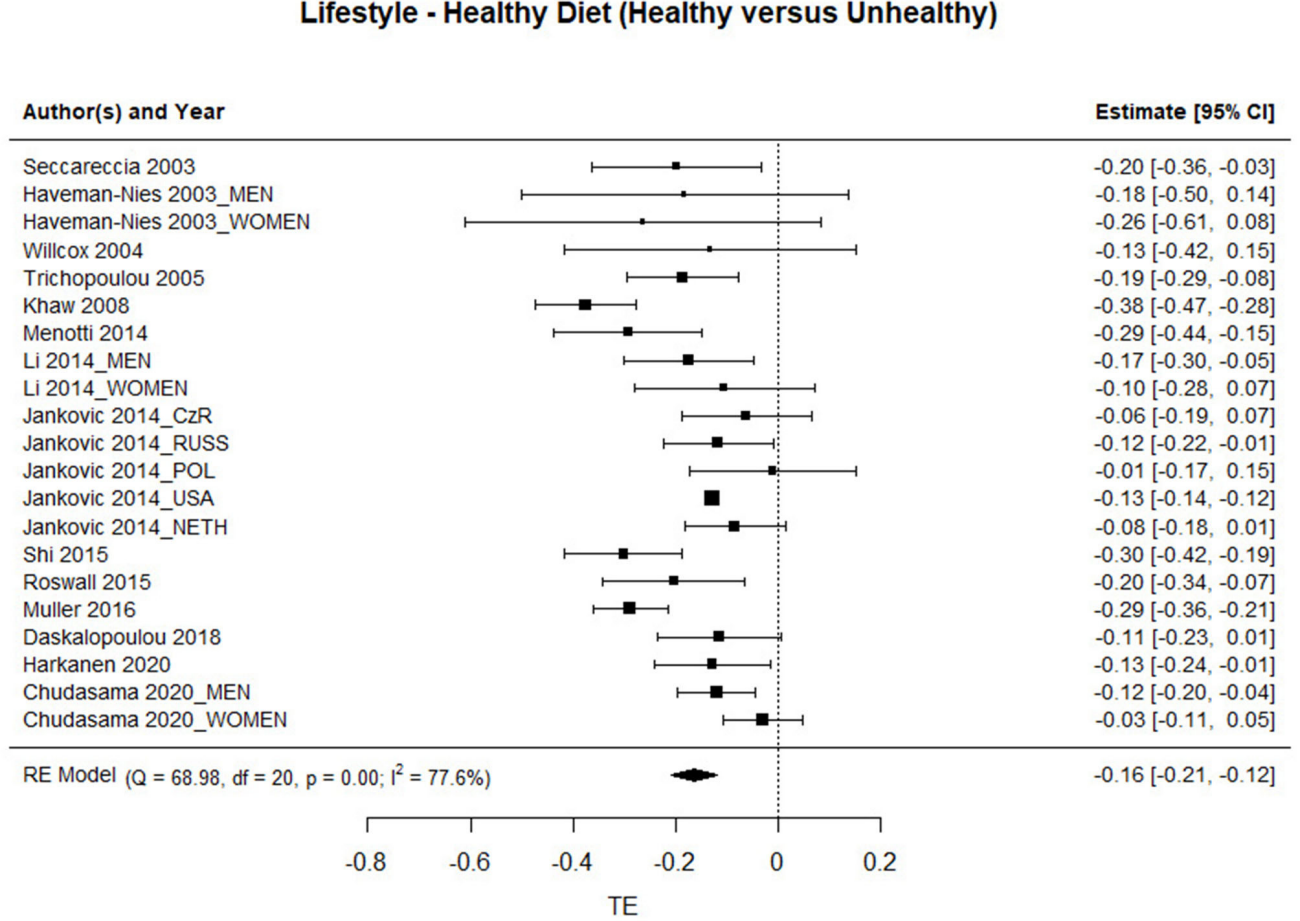
Association between having a healthy diet and the risk of mortality.

The forest plot with the studies involved in the healthy diet factor are presented in Figure 4. The results show a significant association between having a healthy diet and mortality [HR = 0.849; 95%CI: 0.812–0.888], which means that people who follow a healthy diet have a mortality rate of 15% less than those who have an unhealthy diet. Heterogeneity in this factor is also high [Q_Diet_(20) = 68.978, *p* < 0.001].

### Sleep 7-8 Hours

**Figure 5:**
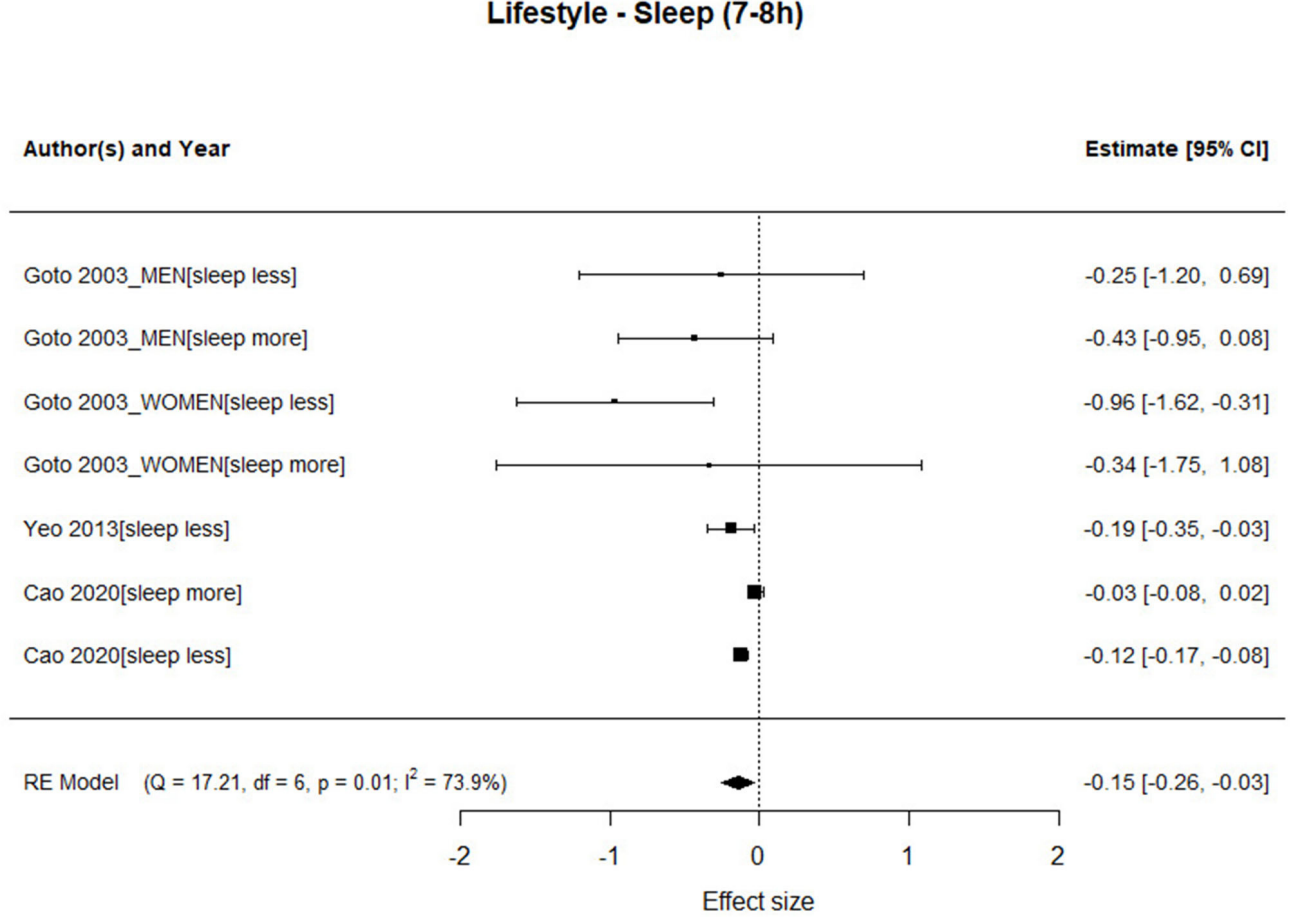
Association between hours of sleep and risk of mortality.

Figure 5 shows the forest plots with the studies that provide results on sleeping 7 or 8 h/day. People who slept 6 h or less or those who slept 9 h, or more were set as the control group. The results show a significant association between sleeping 7 or 8 h and mortality [HR = 0.87; 95%CI: 0.772–0.969], specifically people who sleep from 7 or 8 h/day have an associated mortality risk of about 13% less than people who have the habit of sleeping 9 h or more or 6 h or less. Regarding the heterogeneity of the studies analyzed in this factor, a high value was observed [Q_Sleeping_(6) = 17.207, *p* < 0.001].

### Weight Control

**Figure 6:**
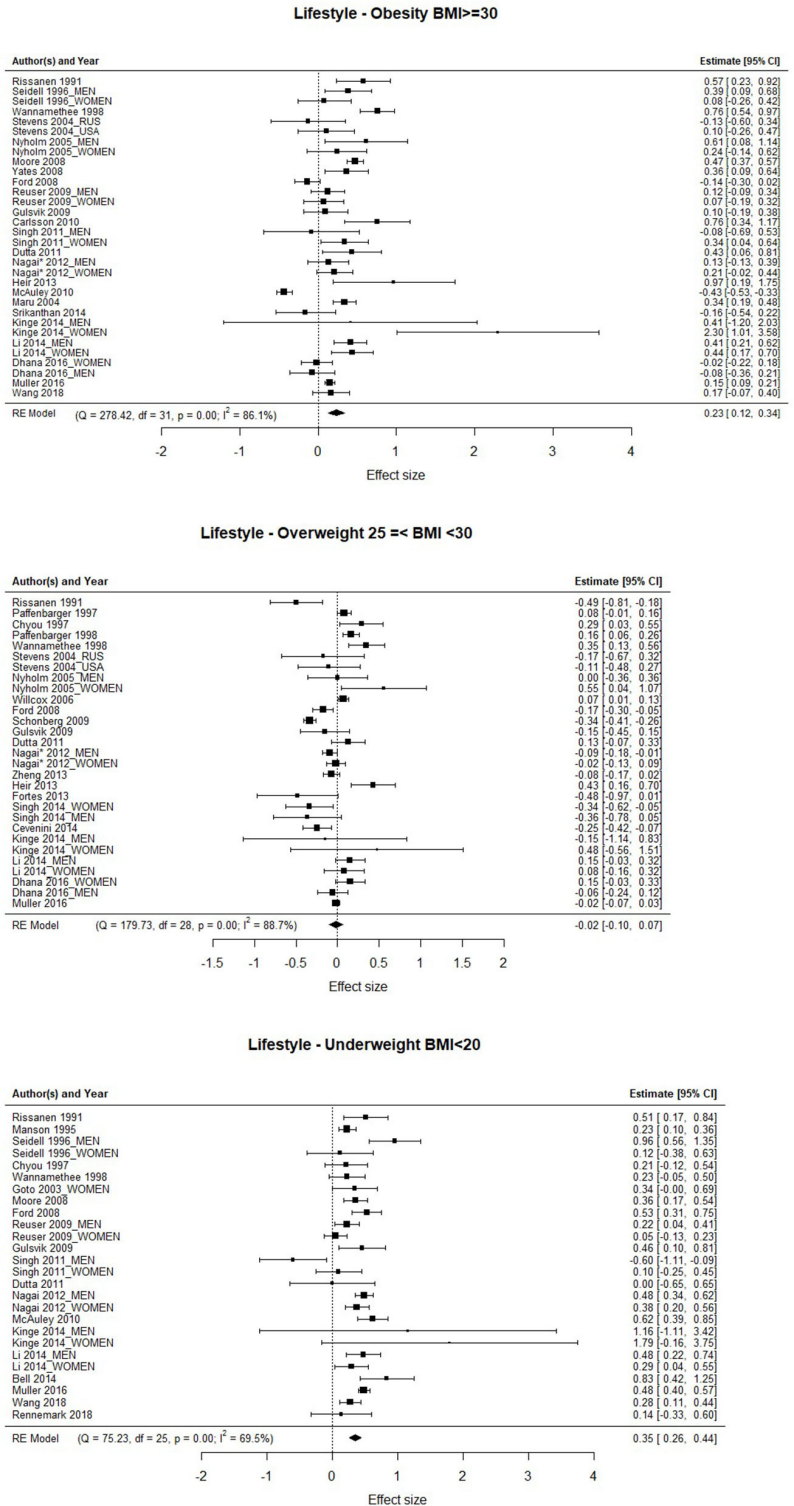
Association between obesity, overweight, and the risk of mortality.

Body mass index was considered as a proxy for weight control. The forest plots with the three groups of BMI are shown in Figure 6. The three cases of BMI (Obesity, Overweight, and Underweight) are analyzed taken the participants with normo-weight as the comparison group. The obesity group shows a significant association with mortality [HR = 1.26; 95%CI: 1.132–1.405], in which the mortality rate is significantly higher than in the normo-weight group. Similar results have been found with studies in which the focus group is Underweight [HR = 1.42; 95%CI: 1.296–1.594], in that case there is a significantly higher mortality rate associated with the underweight. However, there is not significant association between Overweight and mortality [HR = 0.984; 95%CI: 0.906–1.067]. The heterogeneity along the three focus group is significant between the studies [Q_obesity_(31) = 278.422, *p* < 0.001; Q_overweight_(28) = 179.732, *p* < 0.001; Q_underweight_(25) = 75.228, *p* < 0.001].

### Analysis of Moderators

Four variables (mean of participant's age at the baseline of the study, follow-up years of the study, gender, and year of publication) were analyzed to evaluate the role of potential moderators. Obviously, we have not analyzed the potential role of moderators in those factors whose effect is not significant. That is, it was analyzed for all factors except overweight. Table 2 shows the results of the meta-regression models. A first inspection of the table reveals that very few of the tests yield significant results. First, there are not any moderating variables that reaches statistical significance for the physical activity factor; therefore, we can conclude that there is no evidence that the association between the mortality rate and the three first ways of quantifying physical activity (PA Yes-No, PA Frequency and Vigorous PA) is moderated by these variables. However, the results show that the gender of the participants has a moderator role on the association between the mortality rate and regular walks. The negative slope reflects that the association is lower as smaller is the percentage of women in the sample. In other words, regular walk is more important for survival of women than for men.

**Table 2:** Analysis of publication bias associated with significant estimates.

**Factors**	**N_**fs**_**	**RC**	**Rank^T^**	**Egger^T^**	**Trim and Fill**	
					**Imputed values**	**Corrected estimation**
PA (Yes–No)	2,127	90	0.690	0.549	0	HR = 0.75 (0.672–0.831)
PA frequency	916	85	0.675	0.361	2	HR = 0.76 (0.633–0.903)
Vigorous PA	8,923	205	**0.001**	0.099	3	HR = 0.69 (0.648–0.739)
Walking	810	100	**0.022**	**0.000**	4	HR = 0.76 (0.701–0.814)
Leisure activity/leisure physical activity	325	75	0.153	**0.005**	1	HR = 0.82 (0.729–0.917)
Healthy diet	2,058	115	0.455	0.793	0	HR = 0.85 (0.812–0.888)
Sleeping	70	65	1.000	**0.021**	4	HR = 0.90 (0.822–0.995)
Obesity	1,028	190	0.618	**0.013**	4	HR = 1.19 (1.055–1.337)
Underweight	1,897	145	0.931	0.774	1	HR = 1.41 (1.292–1.545)

*Egger' Asymmetry Test and the rank correlation test p-value < 0.05 are in bold. RC, Rosenthal's criterion; PA, physical activity; BMI, body mass index*.

T * p-Value for Egger' Asymmetry Test and the rank correlation test*.

In addition, the studies involved in the “PA Vigorous” dimension reported measures based on the PA intensity in some cases, and measures referred to the PA quantity in other cases. When the intensity of the physical activity was informed, it usually was measured in METs (PA is estimated as the energy cost of a given activity divided by resting energy expenditure), and the PA quantity was linked to the number of hours per week, hours per month or kilocalories consumed for the subject during a regular physical activity. For this reason, two categories were considered, one called *INTENSITY*, and another called *QUANTITY*. The objective was to analyse a potential moderator effect. The analysis of the “Intensity-Quantity” moderator showed a no significant effect (Slope = −0.034, *p* > 0.10, 95% CI [−0.17 to 0.10]) therefore it is possible to conclude that the disaggregation does not evidence any difference. Based on this result, the “PA Vigorous” group is proposed as a unique PA dimension.

On the other hand, the association between the mortality rate and frequently engaging in leisure time activities, including or not physical activities, is moderated by the average age of participants at baseline. In other terms, performing leisure time activities has less relation to the mortality rate (the association is lower) the older the person is at the beginning of the study.

For the Sleeping factor, the length of the follow-up interval and the year of publication of the study are moderators of the association between the mortality rate and sleep 7 or 8 h. In the case of follow-up years, the negative slope indicates that the association is smaller the more years it is followed during the study. The slope is positive for the year of publication and indicates that the association is greater in the most recent studies.

Regarding the BMI, the only significant moderator of the association between the mortality rate and overweight is the length of the follow-up years of the study, which shows a positive slope, which means that the association is greater the greater the follow-up years. It should be noted that the association between overweight and the rate of mortality was not significant.

The results of the moderators should be interpreted cautiously, since the high number of tests (40) predicts that, assuming a significance threshold of α = 0.05, in the absence of any effect 5% of the tests will be significant (approximately 2, in this set of tests). In this case there are more significant results (6; 15%), but some of them are probably mere type I errors.

### Publication Bias

The well-known tendency to facilitate the publication of significant results over non-significant ones can produce an over-estimation of the effect size, or even the appearance of an artificial, non-existent effect. We have evaluated the degree to which this anomaly, known as publication bias, could be a potential threat to the results of this meta-analysis. In this respect, we have made visual inspection of the funnel-plot figures and we have tested for asymmetry with the Egger's test, the rank correlation test, and the Trim and Fill method. We have also calculated the fail-safe numbers for those effects that are significant. The results are summarized in Table 3.

**Table 3:** Results of meta-regression models on lifestyle factors.

**Focal group**		***k***	**Slope**	**95%CI**
PA (Yes–No)	MAB	16	−0.0033	−0.014 to 0.0074
	FUY	15	0.0071	−0.0041 to 0.0182
	GENDER	16	−0.0012	−0.0049 to 0.0025
	PUBY	16	0.0024	−0.0126 to 0.0175
PA frequency	MAB	13	−0.0029	−0.0163 to 0.0106
	FUY	13	−0.0082	−0.0276 to 0.0113
	GENDER	12	0.0000	−0.0056 to 0.0055
	PUBY	13	−0.0053	−0.0267 to 0.0162
Vigorous PA	MAB	34	0.0023	−0.0038 to 0.0084
	FUY	33	0.0013	−0.0058 to 0.0084
	GENDER	35	−0.0007	−0.0017 to 0.0017
	PUBY	36	0.0018	−0.0081 to 0.0117
Walking	MAB	13	−0.0031	−0.0115 to 0.0052
	FUY	10	0.0417+	−0.0046 to 0.088
	GENDER	13	−0.0017^*^	−0.003 to −0.0004
	PUBY	13	−0.0038	−0.0149 to 0.0072
Leisure activity/leisure physical	MAB	11	−0.0104^*^	−0.0202 to −0.0001
activity	FUY	11	0.0154+	−0.0011 to 0.0319
	GENDER	11	−0.0032	−0.0085 to 0.0028
	PUBY	12	−0.0038	−0.0257 to 0.0116
Healthy diet	MAB	21	−0.0013	−0.0055 to 0.0029
	FUY	21	−0.003	−0.008 to 0.0021
	GENDER	21	−0.0004	−0.0013 to 0.0016
	PUBY	21	0.0074+	−0.0013 to 0.0161
Sleeping	MAB	7	0.0044	−0.0024 to 0.0111
	FUY	7	−0.1657^**^	−0.2887 to −0.0427
	GENDER	7	−0.0000	−0.0077 to 0.0068
	PUBY	7	0.0242^*^	0.0057 to 0.0428
Obesity	MAB	29	−0.0094+	−0.0198 to 0.0010
	FUY	32	0.0150	−0.0031 to 0.0330
	GENDER	32	0.0006	−0.0019 to 0.0030
	PUBY	32	−0.0136 +	−0.0294 to 0.0022
Overweight	MAB	29	−0.0094+	−0.0198 to 0.001
	FUY	28	0.0101^*^	0.0009 to 0.0193
	GENDER	29	−0.0018 +	−0.0036 to 0.000
	PUBY	29	−0.0047	−0.0168 to 0.0075
Underweight	MAB	23	−0.0051	−0.0117 to 0.0014
	FUY	25	−0.0071	−0.0266 to 0.0123
	GENDER	26	−0.0011	−0.003 to 0.0009
	PUBY	26	0.0007	−0.0114 to 0.0128

*MAB, mean participant's age at baseline of the study; FUY, follow-up years of the study; GENDER, female percentage; PUBY, publication year of the study*.

*^+^p < 0.10*;

* *p < 0.05*;

** *p < 0.01*.

*Coefficients in bold are significant (p < 0.05)*.

The table shows that all the results are safe from this threat, in the sense that, although the effect may have been over-estimated, at least it exists and is not a by-product of selective publication, as all fail-safe numbers exceed Rosenthal's criterion. Asymmetry tests reveal no evidence of publication bias. The Trim and Fill test has imputed several values to some of the funnel plot. However, in no case has the estimate ceased to be significant after the imputation of values.

## Discussion

The results of the present meta-analysis allow us to reliably conclude that lifestyles are associated with mortality (survival). The four factors studied, which are usually identified as healthy lifestyles, allow us to predict greater survival. Specifically, doing regular physical activity, engaging in leisure activities, sleeping 7–8 h a day, and staying outside the BMI ranges considered as underweight or obesity are habits that each separately has a greater probability associated with survival after a period of several years.

In all the analyses carried out, we have detected high and statistically significant levels of heterogeneity. This means that in this field of research, like any other that studies complex, high-level constructs, the levels of control over variables are low. Longevity is associated with multiple factors, both environmental and genetic, whose isolated and interacting effects have combined impacts that are very difficult to isolate. Primary studies identify factors with significant associations, but they are probably multi-moderated effects. Furthermore, the operationalization of the factors is very diverse and not always clear, in addition to being frequently based on self-reports that are not always sufficiently reliable. Let us think of the varied operationalizations that have been used of walking or diet, as well as the problems of fidelity of memory about them. The fact that significant factors appear despite these difficulties is remarkable. Nevertheless, we must not forget that this high level of heterogeneity does not allow us to accurately predict the results of future similar studies.

### Regular Physical Activity

Regarding regular physical activity, we analyzed 61 studies which were operationalized and quantified differently. Therefore, our classification includes several possible measures used in the studies reviewed: *Physical activity, Walking*, and *Leisure activities* or *Leisure physical activities*. Based on our findings, all these categories showed that physical activity is an important factor associated with a lower rate of mortality, in all groups.

In our study, *walking* (one of the most common types of activity among older adults) in populations with low activity, regular weekly walks would reduce mortality by 28% within a period of about 13 years in the average. Our results are similar to previous studies that have shown that high intensity walking reduces all-cause mortality (Hamer and Chida, 2008a; Woodcock et al., 2011; Colpani et al., 2018). However, the results showed that gender has a moderator role on the association between mortality rate and regular walks. Women live longer than men; thus, identifying the moderator role of gender is particularly important. Our results support the conclusions in the meta-analysis of Colpani et al. (2018), which underlined that moderate physical activity reduces the risk for adverse health outcomes in women, and previous studies that associated walking with reduced risk of cardiovascular disease incidence and all-cause mortality in women (Hamer and Chida, 2008b).

Furthermore, in this meta-analysis the median of the age is 64.5 years, thus the menopause may play a central role. Before the menopause, estrogen protects the female cardiovascular system through multiple mechanisms, but after menopause, the decline in estrogen levels may be harmful (Muka et al., 2016). Also, as several studies have revealed, physical activity can modify levels of endogenous sex hormones in women, including sex hormone levels (Bjornerem et al., 2004; Ennour-Idrissi et al., 2015).

Our results show people who frequently *engage in leisure time activities and/or leisure physical activities* have a 19% lower mortality risk than people who rarely or never engage in those activities. A previous meta-analysis (Woodcock et al., 2011), which included studies that involve more than 1,000,000 people, found a 24% reduction in all-cause mortality between the least and most intensive physical activity groups. Recent data suggest that this physical activity helps to preserve telomere length. In the study by Cherkas et al. (2008), participants who were less physically active during their leisure time were shown to have shorter telomere lengths relative to subjects performing regular exercise, with a difference that corresponds approximately to 10 years. Nevertheless, in our study, the association between the mortality rate and frequently engaging in leisure time activities, including physical activities, is moderated by the average age of participants at baseline and the years of follow-up of the study. Performing leisure time activities is less related to the mortality rate (the association is lower) the older the person is at the beginning of the study.

Physical activity has been shown to enhance the immune function mainly in less fit subjects or the sedentary population (Romeo et al., 2010), maintaining physiological functions and preserving functional reserve in elderly, reducing the risk of cardiovascular diseases, stroke, hypertension, type 2 diabetes, obesity, and anxiety and depression (Gremeaux et al., 2012). Some possible mechanisms underlying the positive effects of being physically active on aging are the improvement in psychological well-being, control of cardiovascular risk factors (Hu et al., 2000), maximal oxygen uptake (Sagiv et al., 2010), improvement in skeletal muscle function and bone health (Carter and Hinton, 2014).

### Healthy Diet

Regarding healthy diet, 14 studies were synthetized. Consistent with previous investigations, this meta-analysis substantiates the protective association among healthy diet and mortality. Following a healthy diet has a mortality rate 15% lower than in those reporting an unhealthy diet.

During recent decades, there has been growing research on the possibly protective role of dietary factors such as antioxidants and other micronutrients (e.g., minerals, polyphenolic compounds, phytoestrogens), generating increased research into diets rich in fruit and vegetables, under the assumption that an increase in their consumption would reduce the incidence of cancer and cardiovascular disease (Ness and Powles, 1997; La Vecchia and Tavani, 1998; Roswall et al., 2015) and a decrease in mortality rate between 7% in different European countries (Trichopoulou et al., 2005) and 18% Swedish women (Roswall et al., 2015). Longitudinal studies with long lasting follow-ups (up to 36 years) have shown that age-adjusted life expectancy varies from 2 years longer in Europe and the United States (Seccareccia et al., 2003; Jankovic et al., 2014) to 14 years in United Kingdom (Khaw et al., 2008).

### Sleep

Hours of Sleep is an important factor in predicting not only the quality of sleep but also health and survival. Our results show sleeping 7–8 h/day is associated with a mortality risk of 13% less than people who normally sleep 9 h or more or 6 h or less, supporting previous research that found associations between inappropriate sleep duration and mortality, cardiovascular disease and general health in middle age (Yeo et al., 2013; Cai et al., 2015) and in very old adults (Cao et al., 2020).

As some authors have suggested, this association might be due to sleep deprivation causing alterations in cortisol secretion and altered growth hormone metabolism (Spiegel et al., 2004) and other biological factors such as levels of leptin and ghrelin that can increase appetite and caloric intake, reduce energy expenditure, and facilitate the development of obesity and impaired glycemic control (Spiegel et al., 2005), and C-reactive protein that causes chronic inflammation (Dowd et al., 2011).

In our analysis, this association is moderated by years of follow-up. The association is smaller the more years it is monitored during the study, perhaps due to changes in the sleeping pattern along the study or to other factors. Furthermore, sleep duration decreases across age (Chaput et al., 2018), thus longer periods of follow up might reflect major changes in sleeping patterns.

### Weight Control

Our findings highlight that being in the BMI ranges considered **underweight** (BMI below 20) **or obesity** (BMI 30.0 and above) is associated with higher mortality rate than the normo-weight (BMI range 20–24.9). Taking into consideration the high and increasing prevalence of overweight and obesity worldwide (Ng et al., 2014; NCD, 2016), this is an important issue to address. Our findings, as in a previous meta-analysis (Di Angelantonio et al., 2016), cover four continents, and as Di Angelantonio et al. (2016) pointed out, the relationship of BMI with mortality was strong and positive in every region studied. That said, being underweight is not healthy either, particularly when it is a consequence of weight loss from disease processes, thereby representing a higher risk of death (Bales and Ritchie, 2002; Greenberg et al., 2007).

In contrast to previous meta-analyses, our study did not find a significant association between overweight (BMI ranges 25–28) and mortality, suggesting the protective metabolic effects of increased body fat. Furthermore, Zajacova et al. (2011) found that overweight adults experienced lower overall mortality than those who are underweight, normal-weight, or obese. As Keith et al. (2013) pointed out, a possible hypothesis might be that healthy people have a tendency to gain a little weight, while less healthy people have a tendency to lose weight. Researchers suggest a “U-shaped relation between BMI and mortality risk as a result of confounding by preexisting disease (sometimes referred to as reverse causality) or by sarcopenia (loss of lean body mass), typical among the elderly” (Zajacova et al., 2011, p. 430).

### Strengths and Weaknesses

The present meta-analysis includes large published studies representing in total more than 2,800,000 people. The analyses included study populations from Europe, North, Central and South America, Europe, United Kingdom, Asia, and Australia. However, the studies were highly heterogeneous in their methods, with different ways of operationalizing and quantifying the variables of interest. Regardless, the categories of the studies were based on those measures, trying to create a coherent and enlightening classification for each lifestyle studied. Also, results were in line with those of other meta-analyses.

Regarding the comments in the introduction referring to the methodology, and specifically the healthy lifestyles measures, our analysis does not include any subjective measures such as satisfaction or even subjective health self-reports (indirect measures). In the four lifestyles analyzed, objective measures are also available that (if analyzed) could have complemented subjective measures: In *Physical Activity* in all its parameters, these include oxygen consumption, heart rate, meters by time, and other target measures; for *Diet*, objective measures include diet outcome, such as cholesterol levels; for *Weight control*, BMI; and finally, for *Sleeping*, polysomnography (PSG).

Although all studies were adjusted for multiple potential moderators, there are likely to remain different factors, such as unrecorded changes in exposure over time given the length of follow up, possible stressful situations, important life events occurring between baseline and follow up, etc., that could substantially affect the results.

## Future Research

Further research should use more objective measures, eliminating the threat of bias caused by systematic differences in healthy lifestyles. In addition to better understanding the relationship between these healthy lifestyles and mortality, researchers should address the differences between: (1) studies carried out with objective measures and (2) studies with self-reported data, and analyse this categorization as a moderator. Does the effect size change, and how much? Response distortion must be controlled when self-reports are taken as measures. There must be a control of two types of distortions to self-reports (Fernández-Ballesteros and Botella, 2007): subject tendency to respond elicited by response format and independently to the self-report and subject's desire to appear with a specific profile.

In sum, perhaps the most critical aspect in meta-analysis is the appropriateness of the methods used by researchers in outcome evaluation and some restriction must be introduced as criteria for inclusion in health evaluation research program.

## Data Availability Statement

The raw data supporting the conclusions of this article will be made available by the authors, without undue reservation.

## Author Contributions

RF-B is the IP of the project. RF-B, MS-I, and JB designed the meta-analysis. EV-L extracted the information from the studies and performed the codification. EV-L and JB designed and performed the statistical analyses. RF-B and MS-I contributed to the interpretation of the results. RF-B, EV-L, and MS-I wrote the manuscript with support from JB. All authors contributed to the final version of the manuscript.

## Funding

This study is one of the objectives of the Research Project granted by the Spanish Ministry of Science and Innovation: Project: PID2019-109761RB-I00.

## Conflict of Interest

The authors declare that the research was conducted in the absence of any commercial or financial relationships that could be construed as a potential conflict of interest.

## Publisher's Note

All claims expressed in this article are solely those of the authors and do not necessarily represent those of their affiliated organizations, or those of the publisher, the editors and the reviewers. Any product that may be evaluated in this article, or claim that may be made by its manufacturer, is not guaranteed or endorsed by the publisher.
