# Supplementary material for: Behavioral Lifestyles and Survival: A Meta-Analysis
Source: Front Psychol. 2022 Feb 4;12:786491. doi: 10.3389/fpsyg.2021.786491 (PMC8854179; doi:10.3389/fpsyg.2021.786491)
Supplement: Supplementary file 1 [file Table_1.docx]

# Supplementary Material Table S1. Summary of studies

| **Author** | **Publication Year** | **Study name** | **Region** | **N at Base line** | **Mean age at Baseline** | **Follow-up length median (years)** | **Sex** | **Factors assessed** | **Adjustments** |
| --- | --- | --- | --- | --- | --- | --- | --- | --- | --- |
| Grand et al. | 1990 | Haute-Garonne Study | France | 645 | 74,4 | 4 | F/M | PA (Yes-No) | Age |
| Rissanen et al. | 1991 | SIIF | Finland | 2731 | 59,5 | 12 | F | BMI | Age and region |
| Rakowski et al. | 1992 | LSOA | USA | 5901 | 77,5 | 4 | F/M | PA, Walking | Physical status (heart, hypertension, Stroke, Cancer, Diabetes, Number ADL, Number IADL, BMI), age, sex, race, education, living arrangement, self-rated health, social involvements. |
| Sherman et al. | 1994 | FS | USA | 1404 | 61,7 | 16 | F | PA Vigorous | Age, systolic blood pressure, serum cholesterol, cigarettes smoked per day, Metropolitan life insurance chart weight, and presence or absence of glucose intolerance, left ventricular hypertrophy, chronic obstructive pulmonary disease, and cancer |
| Manson et al. | 1995 | NHS | USA | 115195 | 42,5 | 16 | F | BMI | Age in five-year categories, smoking, menopausal status, oral-contraceptive and postmenopausal hormone use, and parental history of myocardial infarction before the age of 60. |
| Lissner et al. | 1996 | GS | Sweden | 1036 | 49,0 | 20 | F | PA Vigorous | Adulthood activity index, initial age, and baseline levels of and 6-year changes in smoking, serum triglycerides, BMI, waist-hip ratio, and diastolic blood pressure. |
| Seidell et al. | 1996 | CBPCD | Netherlands | 23351 | NA | 12 | F/M | BMI | Age |
| Paffenbarger et al. | 1997 | HA and UPA | USA | 14787 | 64,5 | 14 | M | PA Vigorous, PA Frequency, BMI | Age, physical activity, cigarette smoking, hypertension, overweight-for-height, alcohol consumption, early parental death, selected chronic diseases. |
| Chyou et al. | 1997 | HHP | USA-Hawaii | 8006 | 56,5 | 22 | M | BMI | Age, FEV, physical activity index, serum cholesterol, systolic blood pressure, alcohol and smoking |
| Paffenbarger et al. | 1998 | HA College | USA | 17815 | 57,5 | 16 | M | PA Vigorous, Walking, BMI | Age, lifestyle factors, BMI, parents dead before age 65 years, chronic disease. |
| Bath et al. | 1998 | NLSAA | UK | 1042 | 75,6 | 12 | F/M | PA Vigorous, Walking | Age, health status, smoking status and weight category |
| Wannamethee et al. | 1998 | BRHS | UK | 7142 | 49,5 | 15 | M | PA Vigorous, BMI | Age, alcohol intake, and BMI |
| Hakim et al. | 1998 | HHP | USA | 707 | 69,9 | 12 | M | Walking | Age, total and HDL cholesterol, hypertension, diabetes, alcohol use, the overall physical-activity index, and preference for a Japanese diet (percentage of foods) |
| Konlaan et al. | 2000 | SASLC | Swedish | 10609 | 47,5 | 13,2 | F/M | Leisure Activity, PA (Yes-No) | Age, sex, protracted disease, smoking, cash buffer, and music-making. |
| Rockhill et al. | 2001 | NHS | USA | 80348 | 46,6 | 14 | F | PA Vigorous, Walking | Age at baseline, smoking status, recent alcohol consumption, height, BMI, and postmenopausal hormone use |
| Seccareccia et al. | 2003 | SCS-CD | Italy | 1536 | 54,7 | 30 | M | Diet | Age, daily energy intake, smoking, physical activity, systolic blood pressure, total cholesterol, BMI, and fruit consumption |
| Goto et al. | 2003 | Ohgimi Village | Japan | 251 | 73,0 | 12 | F/M | PA, Sleeping, BMI | Health behaviour, social activity, age, functional status (ADL, BADL), BMI (only female group), serum albumin, medical history (CVD and Hypertension) only male group. |
| Gregg et al. | 2003 | SOF | USA | 7553 | 76,9 | 10,6 | F | PA Vigorous, Walking | Age, smoking, BMI, stroke, diabetes, hypertension, and self-rated health at baseline. |
| Yu et al. | 2003 | CCHDS | USA | 1975 | 51,5 | 10,5 | M | PA Vigorous | Age and combined light and moderate-intensity activity (logarithmic scale), age, diastolic blood pressure, and BMI as continuous variables, smoking status, social class, family history of CHD among first degree relatives before age 55, history of diabetes mellitus in the past five years, and job physical activity class. |
| Haveman-Nies et al. | 2003 | SENECA | Europe^1^ | 1091 | 72,5 | 10 | F/M | Diet, PA (Yes-No) | No adjustments |
| Willcox et al. | 2004 | HHP | USA | 1915 | 54,7 | 36 | M | Diet | Age, alcohol consumption, physical activity, and macronutrient composition |
| Stevens et al. | 2004 | US-Russia LRC | USA & Russia | 1359 | 69,5 | 17,6 | F/M | BMI | Age, smoking, education, alcohol, and fitness |
| Trichopoulou et al. | 2005 | EPIC (elderly Study) | Europe^2^ | 74607 | 67,8 | 7,4 | F/M | Diet | Sex, age, diabetes mellitus BL, waist to hip ratio, BMI, educational achievement, smoking status, physical activity at work, physical activity at leisure, consumption of potatoes, consumption of eggs, consumption of sugar and confectionery, and total energy intake. |
| Franco et al. | 2005 | FHS | USA | 9181 | 59,3 | 46 | F/M | PA Vigorous | Age, sex, smoking at baseline, and any comorbidity (cancer, left ventricular hypertrophy, arthritis, diabetes, ankle oedema, or pulmonary disease). |
| Fang et al. | 2005 | NHANES-I | USA | 1874 | 38,7 | 17 | F/M | PA Vigorous, PA Frequency | Age, gender, race, BMI, education, history of diabetes, smoking status, alcohol drinking, dietary caloric, sodium, calcium and potassium intake, systolic blood pressure, and serum cholesterol. |
| Stessman et al. | 2005 | JLS | Israel | 461 | 70,0 | 12 | F/M | PA Vigorous | Good vision, volunteer work |
| Nyholm et al. | 2005 | SHCC | Swedish | 533 | 63,1 | 5,4 | F/M | BMI | Age, smoking, and inactive LTPA as covariates |
| Lan et al. | 2006 | NHIS-Taiwan | Taiwan | 2113 | 73,1 | 2,2 | F/M | Leisure Activity, PA Vigorous | Age, sex, education, number of diseases, alcohol use, smoking, body mass index, self-rated health, physical function, and occupation |
| Willcox et al. | 2006 | HHP/HAAS | USA | 5820 | 54,0 | 40 | M | BMI | Age |
| Talbot et al. | 2007 | BLSA | USA | 293 | 76,7 | 10,2 | F/M | PA Vigorous | Cholesterol, BMI, smoking and hypertension were also included in the initial model. |
| Khaw et al. | 2008 | EPIC | UK | 20244 | 58,1 | 11 | F/M | Diet, PA (Yes-No) | Age, sex, and BMI |
| Ford et al. | 2008 | ALSWH-Older Cohort | Australia | 12422 | NA | 9 | F | PA Vigorous, BMI | Self-rated health, smoking status, comorbidity score, BMI, marital status, age. |
| Landi et al. | 2008 | ilSIRENTE Study | Italy | 248 | 85,9 | 2 | F/M | Walking | Age, gender, functional and cognitive disability, congestive heart failure, hypertension, osteoarthritis, depression, number of medications, BMI, cholesterol, and C reactive protein. |
| Moore et al. | 2008 | BCDDP | USA | 50186 | 62,6 | 10 | F | BMI | Race, annual household income, education, Smoking history and physical activity. |
| Yates et al. | 2008 | PHS | USA | 2357 | 72,0 | 25 | M | BMI | Age, smoking status, alcohol intake, exercise frequency, hypertension, diabetes, hypercholesterolemia, angina, and treatment assignment. |
| Byberg et al. | 2009 | ULSAM | Sweden | 2205 | 50,0 | 35 | M | PA Vigorous | Smoking status, obesity status, height and weight, self-perceived health, physical activity at work, diabetes mellitus, and any musculoskeletal, neurological, or psychiatric disorders, and alcohol use. |
| Gulsvik et al. | 2009 | BCBPS | Norway | 788 | 70,0 | 27,5 | F/M | PA (Yes-No), BMI | Gender, BMI, cholesterol, hypertension, smoking, poor socioeconomic, diabetes, CVD, obstructive pulmonary disease, and physical appearance. |
| Stessman et al. | 2009 | JLS-Cohort | Israel | 457 | 70,0 | 18 | F/M | PA Vigorous, PA Frequency | Sex, financial status, origin, BMI, smoking pack-years, ease of performance in activities of daily living, hypertension, ischemic heart disease, diabetes mellitus, history of neoplasm, and renal disease |
| Schonberg et al. | 2009 | NHIS | USA | 16077 | 74,6 | 5 | F/M | BMI | Age, sex, smoking status, comorbid conditions, overnight hospitalization, perceived health and functional measures (IADL, walking several blocks) |
| Reuser et al. | 2009 | HRS | Netherlands | 7195 | 66,6 | 9 | F/M | BMI | Smoke and education |
| Feng et al. | 2010 | CLHLS | China | 16020 | 72,5 | 3 | F/M | PA (Yes-No) | Sex, ethnicity, and age, living environment (urban, town, or rural residence), social support, health status, chronic diseases, lifestyle, weight. |
| Sun et al. | 2010 | NHS | USA | 13535 | 60,0 | 14 | F | PA Vigorous, Walking | Age at BL, education, marital status, if married, husbands’ education, postmenopausal hormone use, smoking status, family history of heart disease, diabetes, cancer, dietary polyunsaturated to saturated fat ratio, intakes of trans fat, alcohol, and cereal fibres (all in quintiles), and intakes of fruits and vegetables and red meat (in terciles). |
| Carlsson et al. | 2010 | Stockholm County Study | Sweden | 935 | 50,5 | 26 | F/M | BMI | Age, sex |
| Lin et al. | 2011 | Tainan city Study | Taiwan | 876 | 72,0 | 8 | F/M | Leisure Activity, PA Vigorous | Age, gender, level of education, habitual smoking and drinking, living status, BMI, cancer, stroke, heart disease, diabetes, liver disease, renal disease, pulmonary disease, hypertension, and osteoarthropathy. |
| Singh et al. | 2011 | AHS | USA | 148 | 87,0 | 17 | F/M | BMI | Education, physical activity, alcohol use, and meat intake. |
| Dutta et al. | 2011 | Iowa EPESE | USA | 2790 | 73,0 | 12,5 | F/M | BMI | Age at Baseline, Sex, and Smoking |
| Xue et al. | 2012 | WHAS II | USA | 436 | 74,0 | 12 | F | PA Vigorous | Age, race, and educational level. |
| Schultz-Larsen et al. | 2012 | BHS | Denmark | 591 | 79,0 | 8,3 | F/M | PA Vigorous | Cognitive impairment, BMI, mobility, co-morbidity, depressive symptoms, education, and living alone. |
| Chakravarty et al. | 2012 | UPA | USA | 2327 | 68,0 | 15,6 | F/M | PA (Yes-No) | NA |
| Gulsvik et al. | 2012 | BCBPS | Norway | 788 | 65,0 | 42 | F/M | PA (Yes-No) | Gender, age, smoking, socio-economic status, hypertension, cholesterol, diabetes, forced expiratory volume (FEV1), and BMI |
| Moore et al. | 2012 | NCICC | NA | 654827 | 61,0 | 10 | F/M | PA Vigorous | Gender, alcohol consumption, education, marital status, history of heart disease, history of cancer, BMI, and smoking status. |
| Buchman et al. | 2012 | MAP | Russia | 893 | 83,0 | 4 | F/M | PA Vigorous | Age, sex, and education, late life social, cognitive activity (7 cognitive activities), chronic health conditions, depressive symptoms. |
| Nagai* et al. | 2012 | Ohsaki NHI | Japan | 20066 | 58,8 | 13 | F/M | BMI | Age groups, smoking status, alcohol drinking, sports, and physical exercise, time spent walking, and education. |
| Edjolo et al. | 2013 | PAQuid | France | 1060 | 78,5 | 20 | F/M | PA (Yes-No) | Satisfaction with level outcome, household tenure, professional help, comfortable housing, years of living in the dwelling, living arrangements, smoking, cognition, recent hospitalisation, diabetes, dysphonia, cardiovascular symptoms, subjective health, educational level, types of house guests, number of relatives, depressive symptomatology, Parkinson’s disease (only male group), limitation due to health status, and group activities (only female group). |
| Yeo et al. | 2013 | KMCC | Korea | 13164 | 54,9 | 9,4 | F/M | Sleeping | Age, sex, educational attainment, BMI, cigarette smoking, alcohol consumption, past history of hypertension, type 2 diabetes, CVD, and metabolic syndrome. |
| Lee et al. | 2013 | ACL | USA | 624 | 77,4 | 6 | F/M | Leisure Activity, PA Frequency Walking | Gender, age, race, income, marital status, smoking. |
| Fortes et al. | 2013 | Roma-III | Italy | 152 | 80,0 | 7,6 | F/M | Walking, BMI | Sex, age, education, BMI, chronic disease, smoking status, tertile cells subsets. |
| Zheng et. al | 2013 | HRS | USA | 9538 | 56,0 | 16 | F/M | BMI | Sex, race/ethnicity, marital status, educational level, income, smoking status, physical activities, activities of daily living limitations, angina, heart failure or heart attack, arthritis, bronchitis or emphysema, cancer, diabetes, stroke, bone fracture, and self-rated health. |
| Heir et al. | 2013 | Five companies in Oslo | Norway | 451 | 55,9 | 32,5 | M | BMI | Non-smoker group, it was adjusted for age, Fitness, cholesterol, SBP |
| Menotti et al. | 2014 | SCS | Italy | 1564 | 54,5 | 40 | M | Diet, PA Vigorous | Age, prevalence of CVD |
| Hamer et al. | 2014 | ELSA | England | 10426 | 65,0 | 7,8 | F/M | PA Vigorous | Age, gender, marital status, and socioeconomic position, self-reported doctor diagnosed chronic diseases (hypertension, diabetes, heart disease, stroke, cancer, chronic obstructive pulmonary disease, and nervous and psychiatric problems), smoking, and depressive symptoms |
| Li et al. | 2014 | EPIC (Heidelberg-Germany) | Germany | 10235 | 51,9 | 11 | F/M | Diet, Leisure Activity, BMI | Education and self-reported hypertension and hyperlipidaemia. |
| Bell et al. | 2014 | HHP | USA | 1292 | 75,7 | 21 | M | PA Vigorous, Walking, BMI | Marital status, BMI (<19), forced expiratory volume in 1 second, diastolic blood pressure, haematological and biochemical variables (fibrinogen, haemoglobin), smoking, alcohol consumption, physical activity index, blocks walked per day. |
| Jankovic et al. | 2014 | HAPIEE, NIH-AARP, RES | Europe^3^ and USA | 354371 | 60,0 | 9,6 | F/M | Diet | Sex, educational level, smoking status, energy intake, alcohol consumption, and physical activity level |
| McAuley et al. | 2014 | VAMC and VAPA | USA | 12417 | 57,0 | 7,8 | M | BMI | Age, ethnicity, examination year, test site, cardiovascular disease (CVD), hypertension, dyslipidaemia, diabetes mellitus, current smoking, CVD medications and fitness (as a continuous variable in metabolic equivalents (1 MET = 3.5 mL/kg/min). |
| Maru et al. | 2014 | DOM | Netherlands | 20555 | 56,8 | 17,4 | F | BMI | Age at baseline (continuous), smoking changes, and weight change. |
| Kinge et al. | 2014 | HALS-1 | Great Britain | 1832 | 58,0 | 12,1 | F/M | BMI | Socioeconomic tertile (SES 0 = most deprived group), Interaction BMI*SES, Age, Interaction Age*BMI. |
| Srikanthan et al. | 2014 | NHNES-III | USA | 3659 | 69,0 | 13,2 | F/M | BMI | Sex, race, central obesity, current smoking, past smoking, cancer, C-reactive protein categories, hypertension, low high-density lipoprotein cholesterol, total cholesterol, HOMA-IR, glycosylated haemoglobin, diabetes, pre-diabetes, and serum creatinine. |
| Singh et al. | 2014 | AHS-II | USA & Canada | 16443 | 53,2 | 5,7 | F/M | BMI | Age, smoking, diet pattern, vigorous physical activity |
| Cevenini et al. | 2014 | GEHA | Italy | 1160 | 93,1 | NA | F/M | BMI | Age and recruitment centre |
| Zhao et al. | 2015 | NISSIP | Japan | 1239 | 64,5 | 10 | M | Walking | Survey year, marital status, work status, education, smoking, drinking status, BMI, regular exercise, daily sleeping duration, regular sports, hypertension, hyperlipidaemia, diabetes mellitus, chronic bronchitis, neuralgia, osphyalgia, arthritis, gait speed, and GDS-15 and TMIG scores. |
| Shi et al. | 2015 | CLHLS | China | 8959 | 92,3 | 4,3 | F/M | Diet, PA (Yes-No) | Age, gender, residence area, smoking, job before 60 years of age, alcohol consumption, physical activity (regular exercise), number of chronic diseases, frequency intake of fruit, vegetable, meat, fish, tea and other |
| Roswall et al. | 2015 | WLHS-SW | Swedish | 44961 | 39,0 | 21,3 | F | Diet | Age, smoking status, duration, current tobacco consumption, time since smoking cessation, school education, BMI, alcohol intake, red meat intake, processed meat intake, energy intake. |
| Keadle et al. | 2015 | NIH-AARP Diet and Health | USA | 165087 | 60,5 | 6,6 | NA | PA Frequency | Age, gender, education, smoking history, history of heart disease, other chronic conditions, health status, BMI. |
| Schnohr et al. | 2015 | CCHS | Australia | 1511 | 40,9 | 11 | F/M | PA Vigorous, PA Frequency | Age, sex, smoking, alcohol intake, education, and diabetes |
| Lee et al. | 2016 | WHI | USA | 92809 | 63,6 | 10,8 | F/M | PA Vigorous | Age, current weight, education, current employment status, ethnicity, leisure-time physical activity, current smoking status, alcohol consumption, general perception of own health, physical functioning score, ever treated for diabetes, history of CVD, cancer, and stroke and all at baseline |
| Muller et al. | 2016 | EPIC | UE | 264906 | 53,1 | 11,5 | F/M | Diet, PA Vigorous, BMI | Age, alcohol intake, smoking, BP, BMI, physical activity, Waist to hip ratio. |
| Aichele et al. | 2016 | MLSC | UK | 6203 | 64,7 | 29 | F/M | Leisure Activity | No adjustments |
| Dhana et al. | 2016 | RS | Netherlands | 3750 | 69,7 | 12 | F/M | BMI | Age, smoking, cigarettes smoked per day, education level, marital status, physical activity, alcohol use and comorbidities |
| Zhou et al. | 2017 | DFTJ | China | 24606 | 63,0 | 5,1 | F/M | PA Frequency | Sex, age, BMI, marriage, education, smoking status and drinking status at baseline. |
| Schnohr et al. | 2017 | CCHS | Denmark | 12314 | 54,0 | 33 | F/M | PA Frequency | Age, sex, smoking, education, household income, drinking habits and diabetes, and mediators (resting heart rate, cholesterol, systolic blood pressure, blood pressure medication and BMI) |
| LaMonte et al. | 2018 | OPACH | USA | 6382 | 78,6 | 3,1 | F | PA Vigorous | Awake accelerometer wear time (h/d), age, ethnicity, education, current smoking, alcohol intake in past 3 months, age at menopause, self-rated general health, and number of comorbid conditions. |
| Rennemark et al. | 2018 | GRC | Sweden | 8456 | 74,3 | 10 | F/M | PA Frequency, BMI | Age, BMI, smoking, leg strength, MMSE, higher education, living alone. |
| Schnohr et al. | 2018 | CCHS | Denmark | 8577 | NA | 25 | NA | Leisure Activity, PA Vigorous | Age, sex, the weekly volume of all LTPAs, smoking, education, income, drinking habits, and diabetes |
| Daskalopoulou et al. | 2018 | 10/66 DRG | America^4^ | 10900 | 74,2 | 4 | F/M | Diet, PA (Yes-No) | Age, gender, education level, and all other lifestyle behaviour variables |
| Wang et al. | 2018 | CLHLS | China | 8026 | 91,8 | 4,3 | F/M | BMI | Age and gender, residence, education, smoking, alcohol drinking, physical activity, intake of fruit, vegetable, meat, fish, and tea |
| Mok et al. | 2019 | EPIC (Norfolk cohort) | UK | 14599 | 59,0 | 12,5 | F/M | Leisure Activity | Age, sex, smoking status, education level, social class, self-rated health, alcohol intake, energy intake, overall diet quality, medical history at baseline, smoking, alcohol intake, energy intake, diet quality, and medical history at the second clinic visit, as well as period-prevalent heart disease, stroke, and cancer, final physical activity assessment, systolic and diastolic blood pressure, triglycerides, low-density lipoprotein cholesterol, and high-density lipoprotein cholesterol at baseline and at the second clinic visit. |
| Larsson et al. | 2019 | COSM and SMC | Swedish | 34221 | 68,6 | 17 | F/M | Leisure Activity | Age, sex, education, work activity, housework, walking/bicycling, exercise, BMI, smoking status, and pack-years of smoking, history of hypertension, history of hypercholesterolemia, diabetes, alcohol consumption, and mDASH diet score. |
| Chudasama et al. | 2019 | UK-BD | UK | 491939 | 58,0 | 6,9 | F/M | Walking | Age, sex, ethnicity, socioeconomic status, employment status, education level, body mass index, smoking status, alcohol consumption, fruit and vegetable, oily fish, non-oily fish, processed meat, red meat intake, and sedentary time. |
| Harkanen et al. | 2020 | FINRISK | Finland | 35804 | 46,8 | 16 | F/M | Diet, Leisure Activity | Education, parent's myocardial infarction, diabetes, smoking, alcohol consumption, diet, life satisfaction risk factors. |
| Chudasama et al. | 2020 | STROBE | UK | 175380 | 57,0 | 7 | F/M | Diet, PA Frequency | Ethnicity (white, non-white), working status (working, retired, other), deprivation (continuous), body mass index (continuous), sedentary time (continuous) and multi-morbidity |
| Li et al. | 2020 | CLHLS | China | 30070 | 92,7 | 16 | F/M | Leisure Activity | Age, sex, education levels, occupation, co-residence, residence, BMI, and marital status, smoking status, alcohol consumption, regular physical activity, frequent fresh fruit consumption, and frequent vegetable consumption, self-reported diseases diagnosed by a doctor (hypertension, diabetes, heart disease and stroke), activities of daily living (ADLs), cognitive impairment and depressive symptoms. |
| Cao et al. | 2020 | CLHLS | China | 17637 | 92,7 | 10 | F/M | Sleeping | Gender, ethnicity, residence, marital status, education level, economic level, systolic BP, diastolic BP, heart rate, smoking, drinking, exercising, diet, BMI, cognitive function, physical disability, and chronic conditions. |

^1^ Belgium, France, Denmark, Italy, The Netherlands, Portugal, Spain, Switzerland, and Poland; ^2^ Denmark, France, Germany, Greece, Italy, The Netherlands, Spain, Sweden, UK; ^3^ Poland, Czech Republic, Russia, and The Netherlands; ^4^ Cuba, Dominican Republic, Peru, Mexico, and Puerto Rico.

**Study names:** SIIF = Social Insurance Institution of Finland, LSOA = Longitudinal Study of Aging, FS = The Framingham Study, NHS = The Nurses’ Health Study, GS = Gothenburg Study, CBPCD = The Consultation Bureau Project on Cardiovascular Diseases, HA and UPA = UPA = Harvard Alumni and University of Pennsylvania alumni, HHP = The Honolulu Heart Program, HA College = Harvard Alumni, NLSAA = The Nottingham Longitudinal Study of Activity and Ageing, BRHS = The British Regional Health Study, SASLC = Swedish Annual Survey of Living Conditions, SCS-CD = Seven Countries Study (Italian rural areas), SOF = The Study of Osteoporotic Fractures, CCHDS = The Caerphilly collaborative heart disease study, SENECA = Survey in Europe on Nutrition and the Elderly: A Concerted Action, US-Russia LRC = Joint US-Russia LRC First Prevalence Study, FHS = the Framingham Heart Study, NHANES-I = First National Health and Nutrition Examination Survey, JLS = The Jerusalem Longitudinal Cohort Study, SHCC = Skara Health Care center, NHIS = The National Health Interview Survey, HHP-HAAS = The Honolulu Heart Program and Honolulu Asia Aging Study, BLSA = Baltimore Longitudinal Study of Aging, EPIC = European Prospective Investigation into Cancer and Nutrition, ALSWH-Older Cohort = The Australian Longitudinal Study on Women’s Health, ilSIRENTE study = Prospective cohort study performed in the mountain community living in the Sirente geographic area, BCDDP = Breast Cancer Detection Demonstration Project, PHS = Physicians Health Study, ULSAM = Uppsala longitudinal study of adult men, BCBPS = The Bergen Clinical Blood Pressure Survey, HRS = The US Health and Retirement Study, CLHLS = The Chinese Longitudinal Healthy Longevity Survey, AHS = The Adventist Health Study, Iowa EPESE = Established Populations for Epidemiologic Study of the Elderly, WHAS II = The Women’s Health and Aging Study, BHS = the Brønshøj-Husum Study, NCICC = The National Cancer Institute Cohort Consortium, MAP = The Memory and Aging Project, a longitudinal cohort study of aging, Ohsaki NHI = Ohsaki National Health Insurance Cohort Study, PAQuid = The French PAQuid (Personnes Agées Quid) Cohort, KMCC = Korean Multi-centre Cancer Cohort study, ACL = The Americans’ Changing Lives, Roma-III = Casa di Riposo Roma III, ELSA = The English Longitudinal Study of Ageing, HAPIEE = Psychosocial Factors in Eastern European Countries Study, NIH-AARP = The National Institutes of Health–AARP Diet and Health (NIH-AARP) Study from the United States, RES = The Rotterdam Elderly Study, VAMC and VAPA = Veterans Affairs Medical Centre and Veterans Affairs Palo Alto, DOM = Diagnostisch Onderzoek Mammacarcinoom, or Diagnostic Investigation into Breast Cancer, HALS-1 = The Health and Lifestyle Survey, NHNES-III = Third National Health and Nutrition Examination Survey, GEHA = EU funded GEHA project, NISSIP = New Integrated Suburban Seniority Investigation Project, WLHS-SW = The Swedish Women’s Lifestyle and Health Cohort, CCHS = The prospective Copenhagen City Heart Study, WHI = The Women’s Health Initiative observational study, MLSC = The Manchester Longitudinal Study of Cognition, RS = Rotterdam Study, DFTJ = The Dongfeng-Tongji Cohort, OPACH = The Objective Physical Activity and Cardiovascular Health Study, GRC = Geriatric research centres in four Swedish regions (Malmö, Karlskrona, Kungsholmen, and Nordanstig), 10/66 DRG = The 10/66 Dementia Research Group, COSM and SMC = The Cohort of Swedish Men and, UKBR = UK Biobank Resource, FINRISK = Finnish population survey on risk factors, STROBE = The Strengthening the Reporting of Observational Studies in Epidemiology.
