# Supplementary material for: Behavioral Lifestyles and Survival: A Meta-Analysis
Source: Front Psychol. 2022 Feb 4;12:786491. doi: 10.3389/fpsyg.2021.786491 (PMC8854179; doi:10.3389/fpsyg.2021.786491)
Supplement: Supplementary file 2 [file Table_2.docx]

# Supplementary Material Table S2. Healthy lifestyle habits assessment.

| **Study** | **Factor** | **Measures** | **Assessment (Categories)** | **Reference group** | **Instrument validation** | **Questionnaire or examination** |
| --- | --- | --- | --- | --- | --- | --- |
| Grand 1990 | Physical activity (YES-NO) | Physical exercise answers were coded as at least once per month out- or in-doors, or as exercise less often. | (1) Yes = Physical exercise at least once per month out- or in-doors (2) No = Physical exercise less often. | No = Physical exercise less often. | Questionnaire of the Haute-Garonne (France) Study. | Self-administered questionnaire |
| Rakowski 1992 | Physical activity (YES-NO) | Regular exercise routine was assessed by the question Do you follow a regular routine of physical exercise? Response options: Yes, No. | (1) Yes = being on a regular exercise schedule (2) No = without regular exercise schedule | Yes = regular exercise schedule | Self-assessment of Activity and Exercise. | Self-reported questionnaire |
| Konlaan 2000 | Physical activity (YES-NO) | Physical exercise answers were coded as at least once per month out- or in-doors, or as exercise less often. | (1) Yes = Physical exercise at least once per month out- or in-doors (2) No = Physical exercise less often. | No = Physical exercise less often. | Interview survey of the Project | Participants were interviewed by non-medical interviewers |
| Goto 2003 | Physical activity (YES-NO) | Physical exercise answers were coded as Exercise seldom, or Non exercise. | (1) Exercise seldom (2) Non exercise. | Non exercise | Questionnaire was based on the health status Assessment Questionnaire of the Elderly developed by the Tokyo Metropolitan Institute of Gerontology. | Questionnaire |
| Haveman-Nies 2003 | Physical activity (YES-NO) | The Voorrips-score was used including household, sports, and leisure time component. To classify PA, sex-specific tertiles (low-, intermediate- and high-physical activity) were constructed from data for the baseline population. | (1) Inactive = participants from the low-activity tertile (2) Active = participants from the intermediate- and high activity tertiles. | Active | The Voorrips Score | Questionnaire |
| Khaw 2008 | Physical activity (YES-NO) | Habitual physical activity referring to activity during the past year. The first question was related usual physical activity at work. The second question asked about the amount of time spent, in hours per week. A simple index was validated against heart rate monitoring with individual calibration in two independent studies. | (1) Physically inactive (coded as 1) was defined as the mix of both conditions, sedentary job and no recreational activity. (2) No physically inactive (coded as 0) involved any category with activity levels above the latter. | No physically inactive | Questionnaire of EPIC-Norfolk cohort | Self-administered questionnaire |
| Gulsvik 2009 | Physical activity (YES-NO) | Inactivity was defined as physical activity less than Sunday strolls or gardening. The questionnaires included information about habits such as smoking, physical activity, dietary habits in childhood and adulthood. | (1) Physical inactivity (1) Not physical inactivity (Active) | Not physical inactivity | The self-administered Norwegian Respiratory Questionnaire | Self-administered questionnaire |
| Feng 2010 | Physical activity (YES-NO) | Question related to the participant's current situation regarding whether he or she practices regular physical exercise. | (1) Yes = Current exerciser (2) No = Not current exerciser | Not current exerciser | Questionnaire based on international standards and adapted to the Chinese cultural/social context and carefully tested by pilot studies. | Interview |
| Chakravarty 2012 | Physical activity (YES-NO) | The main measure was Inactivity defined as absence of vigorous physical activity that works up a sweat such as including jogging, cycling, brisk walking, swimming, and other sports. | (1) Inactive (absence of vigorous physical activity) (2) Active | Active | Questionnaire of the study | Self-reported questionnaires |
| Gulsvik 2012 | Physical activity (YES-NO) | Six questions with dichotomous (yes/no) answers, evolving around participation in different PA during the past 5 years. The respondents who reported YES to more than one PA-question were categorized according to their highest reported level of activity. The respondents answering NO to all of the six questions were assigned to the low/no activity level. | (1) No/Low PA (2) Any PA, Moderate activity (walking at least 2 km or bicycling at least 5 km at least 3 times/week, walking or bicycling to work for 10 min daily, strolling or gardening) or High activity (performing competing sports, taking regular exercise/gym) | No/Low PA | A standardized interview based on a questionnaire; it was part of The Bergen Clinical Blood Pressure Survey. | The assessment was carried out by trained nurses. |
| Edjolo 2013 | Physical activity (YES-NO) | Question: Do you practice regular physical activity (PA)? Response options were Yes and No. | (1) Yes = Regular PA (2) No = Not regular PA | No regular physical activity | The survey was part of the PAQUID research program. | Face-to-face interviews in the participants' homes. |
| Shi 2015 | Physical activity (YES-NO) | Do you do exercise regularly at present, including jogging, playing ball, running and Qigong? Response options: Yes, No. | (1) Having physical activity (2) No physical activity | No physical activity | Standard questionnaire of the Project | Participants were interviewed in their homes by health workers |
| Daskalopoulou 2018 | Physical activity (YES-NO) | The participant chose a category that represents her/him on How physically active she/he is. The response options were very physically active, fairly, not very, and not at all. | (1) Very or Fairly Active (2) Not very or Never Active | Not very or Never Active | Questionnaire of the 10/66 Dementia Research Group (10/66 DRG) | Self-reported questionnaires |
| Paffenbarger 1997 | Physical exercise / Frequency | Physical activity Index included walking, stair climbing. and sports or recreational activities Physical activities were equated to indices that expressed both quantity (kcal per week) and intensity (METs of effort) | (1) Sedentary = physical activity index < 1500 kcal per week (2) Active = physical activity index >= 1500 kcal per week | Physically active | Physical Activities Questionnaire | Self-reported questionnaires were provided by postal surveys |
| Fang 2005(A) | Physical exercise / Frequency | Do you get much exercise things you do for recreation, or hardly any exercise, or in between? Response options (1) much exercise, (2) moderate exercise (3) little or no exercise. | (1) Least exercise (2) Moderate exercise (3) Most exercise. The index of Moderate exercise was extracted | Least exercise | Physical Activity Questionnaire of the NHANES-I Project | Self-reported questionnaire at baseline of the study |
| Stessman 2009 | Physical exercise / Frequency | 4-item questionnaire of Physical activity status was determined according to the study question: How often are you physically active? The answers were (1) Less than 4 h/wk (2) about 4 h/wk, (3) Regular physical activity (e.g., walking at least an hour daily), and (4) Vigorous sports at least twice weekly (e.g., jogging or swimming). | (1) Less than 4 h/wk (2) >= 4 h/wk or more, (3) Walking daily (4) Vigorous sports twice weekly. The index of Walking daily was extracted. | Less than 4 hours per week | The questionnaire was adapted from the Gothenburg population study of 70-year-olds | Self-reported questionnaire at baseline of the study |
| Lee 2013 | Physical exercise / Frequency | Frequency of engaging in three common types of PA using the following question: How often do you engage in active sports or exercise? Response options were based on 4-point Likert scale. | (1) Never (2) Rarely (3) Sometimes (4) Often. The index of active sports often was extracted | Never active sports | Physical Activity Level questionnaire is part of the Americans’ Changing Lives Longitudinal Study | Self-reported questionnaires |
| Keadle 2015 | Physical exercise / Frequency | Engaging in moderate to vigorous physical activity (MVPA) was assessed by asking about the average time spent each week in the past 10 years in activities of a moderate-vigorous intensity using the following question How often did you participated in moderate and vigorous activities at the following ages and time? Ages categories were (1) 15-18 years old (2) 19-29 years old (3) 35-39 years old (4) In the past 10 years. The frequency options were (1) Never (2) Rarely (3) Weekly, but less than 1-3 h/wk (4) 4-7h/wk (5) More than 7h/wk | (1) MPA <1 h/wk, (2) MPA = 1 to 4 h/wk (3) MPA = 4 or more h/wk. The index of the second category (MPA = 1 to 4h/wk) was extracted. | MPA < 1hr/wk | Risk Factor Questionnaire is part of the NIH-AARP Diet and Health Study. | Self-reported questionnaires |
| Schnohr 2015 | Physical exercise / Frequency | Survey questions: weekly quantity of jogging, frequency of jogging, and the subject’s own perception of pace (slow, average, fast). According to the frequency the categories were defined. | (1) Sedentary non jogger (2) Jogging frequency =1 time/week, (3) Jogging frequency 2 to 3 times/week (4) Jogging frequency >3 times/week. The index of the fourth group was extracted. | Sedentary non joggers | The Copenhagen City Heart Study Leisure Time Physical Activity Questionnaire | Self-administered questionnaire. |
| Schnohr 2017 | Physical exercise / Frequency | The questions was about the PA in leisure time within the last year, and the response options were 4 groups: (1) Sedentary, almost completely sedentary (e.g. reading, watching television or movies) or light physical activity less than 2 h/wk (<3 metabolic equivalents METs) (2) Light physical activity 2 to4 h/wk (3 to 4.5 METs) (3) Moderate physical activity more than 4 h/wk or more vigorous activity for 2 to 4 h/wk (4.5 to 6 METs) (4) High, vigorous physical activity more than 4 h/wk or regular heavy exercise or competitive sports several times per week (>6METs). | (1) Sedentary, almost completely sedentary (<3 METs) (2) Light physical activity (3 to 4.5 METs) (3) Moderate physical activity (4.5 to 6 METs) (4) High, vigorous physical activity (>6METs). The index of Moderate PA was extracted. | Sedentary, almost completely sedentary | The Copenhagen City Heart Study Leisure Time Physical Activity Questionnaire | Self-administered questionnaire. |
| Zhou 2017 | Physical exercise / Frequency | Questionnaire contains five questions: Did you do RPA in the past six month, at least 20 minutes each time? Which type of PA did you do? (Including leisure-time PA, transportation and exercises), How many times did you do on RPA each week? How long did you spend on PA each time? How many years have you done RPA?. Walking, biking, dancing, tai-chi, doing exercise in gym, playing ball games, jogging, swimming, and climbing were included. Individual energy expended was calculated by multiplying estimated MET value by the duration in hours/week. The estimated MET hours/week for RPA were 3 for walking, 4 for biking, 4.5 for tai chi, 7.5 for jogging or swimming, 5 for dancing, 4.5 for climbing, 6 for playing ball games or doing exercise in gym. The overall energy expenditure in MET hours/week across all types of regular physical activities for each subject were summed then. | (1) 0 MET h/wk (2) 0.1 to <7.5 MET h/wk (3) 7.5 to <22.5 MET h/wk (4) 22.5 to <37.5 MET h/wk (5) 37.5 to <75.0 MET h/wk (6) >= 75 MET h/wk. The index of RPA >= 75 MET h/wk was extracted. | RPA = 0 MET h/wk | Regular physical activity (RPA) measures are based on metabolic equivalent task (MET) hours per week. | Self-reported questionnaires collected by trained investigators |
| Rennemark 2018 | Physical exercise / Frequency | Survey question: How often have you been engaged in light physical activity (such as going for walks, taking short bicycle tours, doing light gymnastics, playing golf or engaging in similar activities) during the last 12 months? Response options were 1 = Never, 2 = Once a month, 3 = Two to three times a month, 4 = More than once a week but not every day, 5 = Every day. MPA was dichotomized into either once every month or less, or 2 to 3 times every month or more. | (1) MPA either once every month or less (2) MPA 2 to 3 times every month or more. | MPA either once every month or less | Questionnaire based on the study protocol. | The examinations were performed at geriatric research centres in four Swedish regions. The examinations were carried out by trained research staff |
| Chudasama 2020 | Physical exercise / Frequency | Survey question: In the last 4 weeks, did you spend any time doing the following: walking for pleasure, light DIY (do-it-yourself, i.e., home maintenance and improvement and gardening activities), heavy DIY (e.g., using heavy tools, weeding, lawn mowing, digging, carpentry), strenuous sports (i.e., sports that make you sweat or breathe hard), other exercises (e.g., swimming, cycling, keep fit, bowling), none of the above. Participants could select more than one activity and were asked to quantify their participation by frequency (i.e., number of times in the previous 4 weeks) and duration. The intensity was expressed in terms of standardised metabolic equivalent of task (MET) values. Regular PA was established on >=500 MET-minutes/week. | (1) Regular physical activity >=500 MET-minutes/week (2) No regular physical activity <500 MET-minutes/week | No regular physical activity | The questionnaire is part of the UK Biobank Centre protocol. And the final categorization was established on the basis of the global health recommendations for physical activity. | In-person baseline interview at the UK Biobank centre |
| Sherman 1994 | Physical exercise / Vigorous | Participants were asked to quantify number of hours in an average day spent in each of five activity levels: (1) Sleeping (2) Sedentary (3) Light activity (4) Moderate Activity (5) Heavy activity. Physical activity (PA) index is calculated for sample subject. The measure of PA was the sum of the weighted values, with 24 being the absolute minimum. Then the level of PA of a person was ranked and grouped them into quartiles. | (1) Less active (PAI = 25-30), (2) 2nd quartile (PAI = 31-32), (3) 3rd quartile (PAI = 33-34), (4) Most active (PAI = 35-58). The index of Most active was extracted | Less active | Framingham Study Protocol | 20-minutes survey by a trained physician-interviewer |
| Lissner 1996 | Physical exercise / Vigorous | 4-point scales about the PA during Leisure time were administered with response options were from 1= least active to 4 = most active. That score was thereby estimated for the following periods of subject’s life, ages 20-38 years (all women), age 39 to the present (women aged 46-60), and during the past 12 months (all women). Index of total adulthood activity (adulthood activity index) was calculated for each woman by averaging all her responses, which resulted in a continuous version of the original 4-point scale. | (1) Low (almost completely inactive) (2) Medium (at least 4h p/wk physical activity) (3) High (regular physical activity or regular heavy physical training or competition). The index of High LTPA was extracted. | Low PA (almost completely inactive) | Physical activity questionnaire was adapted but not validated | Questionary was administered verbally by a physician during a general health examination. |
| Paffenbarger 1997 | Physical exercise / Vigorous | Physical activity Index (Kcal/week), the index included walking, stair climbing and sports or recreational activities. | (1) PA = 1500 Kcal/wk or more (2) PA <1500 Kcal/wk. The index of PA <1500 Kcal/week was extracted. | PA = 1500 Kcal/wk or more | Physical Activities Questionnaire | Self-reported questionnaires were provided by postal surveys |
| Bath 1998 | Physical exercise / Vigorous | Customary physical activity (CPA) in later life was assessed through seven categories: outdoor productive categories activities (e.g., car maintenance), indoor productive activities (e.g., housework), walking, shopping, leisure activities (e.g., cycling, swimming), strength activities (e.g. climbing high steps) and joint flexibility activities (e.g. reaching for high shelves). Respondents were divided into 3-groupings according to tertile ranges for the CPA at baseline factor scores (derived from principal component with varimax rotation). | (1) High activity (2) Intermediate activity (3) Low activity. The index of Low CPA was extracted. | High activity | Customary physical activity | The detailed activity inventories were administered by trained interviewers. |
| Paffenbarger 1998 | Physical exercise / Vigorous | Physical activity Index included walking, stair climbing. and sports or recreational activities Physical activities were equated to indices that expressed both quantity (kcal per week) and intensity (METs of effort) | (1) <1000Kcal/wk, (2) 1000 to 1999 Kcal/wk, (3) >=2000 Kcal/wk (). The index of PA >=2000 Kcal/wk was extracted. | PA = <1000Kcal/wk | Physical Activities Questionnaire | Self-reported questionnaires were provided by postal surveys |
| Wannamethee 1998 | Physical exercise / Vigorous | Questions about the pattern of physical activity: (1) regular walking or cycling, such as weekday journeys that included travel to and from work (2) recreational activity includes gardening, pleasure walking and do-it-yourself jobs, (3) sporting activity (vigorous) includes running, swimming, golf, tennis, sailing, and digging. The PA score was based on the frequency and the type (intensity) of PA. Participants were classified according to their total score. | (1) Inactive (2) Occasional (3) Light (4) Moderate (5) Moderately vigorous (5) Vigorous. The index of Vigorous physical activity was extracted. | Inactive | The total score was based on the recommendations of a National Heart, Lung and Blood Institute (NHLBI) workshop and the Minnesota intensity codes | A standard questionnaire was administered by research nurses. |
| Rockhill 2001 | Physical exercise / Vigorous | One question: For how many hours per week, on average, do you engage in activity strenuous enough to build up a sweat? Then measures of cumulative average physical activity level (in units of hours per week) were created and 5 levels were categorized. | (1) Less than 1 h/wk (2) 1 to 1.9 h/wk (3) PA = 2 to 3.9 h/wk (4) PA = 4 to 6.9 h/wk (5) PA = 7 or more hours per week. The index of PA >= 7h/w was extracted | Less than 1 hour per week | Protocol from The Nurses’ Health Study | Participants were nurses and they returned a mailed questionnaire about their medical histories and lifestyles. |
| Gregg 2003 | Physical exercise / Vigorous | Physical activity (PA) was calculated and expressed in kilocalories per week and finally it was divided by quintiles. | (1) PA les than 169 Kcal/wk (2) PA = 163 to 503 Kcal/wk (3) 504 to 1045 Kcal/wk (4) 1046 to 1906 Kcal/wk (5) 1907 Kcal/wk or more. The total physical activity >= 1907 Kcal/wk was extracted | Less than 169 Kcal/wk | A modified version of The Harvard Alumni Questionnaire | Not mentioned |
| Yu 2003 | Physical exercise / Vigorous | Questions involved the type and duration of their leisure activities during the previous 12 months. The energy expenditure expressed as an activity index (AI) in kcal/day was estimated. Light AI was defined by summing those activities having intensity codes 2.0, 2.5, 3.0, 3.5, and 4.0 (for example, walking, bowling, sailing), moderate AI was obtained by summing activities with intensity codes of 4.5, 5.0, and 5.5 (for example, golfing, digging, dancing), and heavy AI was defined by summing all activities having intensity codes > 6.0 (for example, climbing stairs, swimming, jogging). Finally, tertiles for the energy expenditure of heavy intensity activity during leisure time at baseline was calculated and the categories were created. | (1) Tertile 1 = 0.0 to 0.6 kcal/day (2) Tertile 2 = 0.7 to 23.8 kcal/day (3) Tertile 3 = 23.9 to 2142.9 kcal/day. The index of tertile 3 was extracted. | Tertile 1 = 0.0 to 0.6 | A detailed questionnaire derived from the Minnesota LTPA questionnaire | A trained interviewer interviewed subjects |
| Fang 2005(A) | Physical exercise / Vigorous | Question was about recreational exercise: Do you get much exercise things you do for recreation, or hardly any exercise, or in between? | (1) Least exercise (2) Moderate exercise (3) High exercise. The index of Most exercise within the normotensive group. | Least exercise | Questions were part of The National Health and Nutrition Examination Survey (33) | Self-reported survey |
| Franco 2005 | Physical exercise / Vigorous | How long they spent in a typical day at various levels of activity (sleeping, resting, or engaging in light, moderate, or heavy physical activity). The reported levels of activity were weighted based on the estimated oxygen consumption for each activity to reflect metabolic expenditure corresponding to metabolic equivalents. Weights used were as follows: for sleeping=1, for being sedentary=1.1, for light activity=1.5, for moderate activity=2.4, and for heavy activity=5. Finally, a daily physical activity score was calculated by adding the sum of the weighted hours for each level of activity. The minimum possible score was 24 for a participant sleeping 24 hours a day. Based on tertiles of the physical activity score, we grouped the participants into 3 levels | (1) Low (Score < 30) (2) Moderate (Score = 30 to 33) (3) High (Score > 33). The index of High PA was extracted. | Low (Score<30) | Questionnaire from The Framingham Heart Study | No mentioned |
| Stessman 2005 | Physical exercise / Vigorous | Physical activity was defined as at least 1 hour of light activity four times a week or intense activity twice weekly. | (1) PA = 1h per 4 times weekly of light activity or intense activity twice weekly (2) PA < 1h per 4 times weekly of light activity or non-intense activity twice weekly | PA < 1h per 4 times weekly of light activity or non-intense activity twice weekly | No specified | Not mentioned |
| Lan 2006 | Physical exercise / Vigorous | Question: Did you engage in any kind of leisure activity during the last two weeks? Response options were Yes, No. For exercisers, information on names, total times, and durations for each activity (up to three) during the 2-week period was further collected. Then the total amount of energy (kcal per week) was calculated. Energy expenditure (kcal) of each activity per week was obtained by activity intensity code (kcal/min), frequency (times), duration for each time (min)/2. The energy expenditure values were then added up into the total weekly amount of energy expenditure. | (1) Sedentary, (2) <500, (3) 500 to 999, (4) 1000 to 1999, (5) >=2000. The index of PA >=2000 kcal/wk was extracted. | Sedentary | Reliability and Validity were informed in the same publication. | All participants were interviewed with standard questionnaires by well-trained interviewers. |
| Talbot 2007 | Physical exercise / Vigorous | Questions focused on the amount of time spent performing 97 activities, encompassing exercise, housework, and social activities, over the prior 2 years. The time spent in daily activities was based on a typical day, with less commonly performed activities appropriately apportioned. To quantify the intensity of LTPA, each activity was assigned a value for metabolic units (METS, or metabolic equivalents of resting oxygen consumption). The MET unit assigned to the activity was multiplied by the average number of minutes performing each activity in a 24-h period, providing a value for LTPA in MET-min/day. Finally, activities were categorized according to MET intensity. | (1) Low intensity (2) Moderate intensity (3) High intensity. The index of High intensity of LTPA was extracted | Low intensity | The questionnaire was based on The Activity Questionnaire-II (38). The procedures to assig each MET unit to the activity were based on Talbot et al., 2000. | Self-reported questionnaire at the Gerontology Research Centre in Baltimore. |
| Ford 2008 | Physical exercise / Vigorous | Question about the frequency of participation in ‘vigorous’ and ‘less vigorous’ activity was used to derive a PA score. | Exercise level: (1) High (2) Moderate (3) Low (4) None. The index of High Exercise was extracted. | Moderate exercise | Two modified questions from the 1980-1989 Australian National Heart Foundation Risk Factor Prevalence Studies | Self-reported survey |
| Byberg 2009 | Physical exercise / Vigorous | Four questions with Yes-No responses options: (1) Do you spend most of your time reading, watching TV, going to the cinema, or engaging in other, mostly sedentary activities? (2) Do you often go walking or cycling for pleasure? (3) Do you engage in any active recreational sports or heavy gardening at least 3 hours every week? (4) Do you regularly engage in hard physical training or competitive sport? Participants answering yes to question (1) were categorised as Low activity, to question (2) as medium activity, and to oprions (3) and (4) as High activity. | (1) High activity (2) Medium activity (3) Low activity. The index of Low activity was extracted. | High activity | Self-reported Leisure Time Physical Activity | Self-administered questionnaire in survey |
| Stessman 2009 | Physical exercise / Vigorous | 4-item questionnaire of Physical activity status was determined according to the study question: How often are you physically active? The answers were (1) Less than 4 h/wk (2) about 4 h/wk, (3) Regular physical activity (eg, walking at least an hour daily), and (4) Vigorous sports at least twice weekly (e.g., jogging or swimming). | (1) Less than 4 h/wk (2) >= 4 h/wk (3) Walking daily (4) Vigorous sports at least twice weekly. The index of the fourth category was extracted. | Less than 4 hours per week | The questionnaire was adapted from the Gothenburg population study of 70-year-olds | Self-reported questionnaire at baseline of the study |
| Sun 2010 | Physical exercise / Vigorous | Energy expenditure in metabolic-equivalent tasks (METs) measured in hours p/wk was calculated. Each MET-hour is the caloric need per kilogram of body weight p/h of activity divided by the caloric need per kilogram of weight p/h at rest. According to this standard, a MET value of 12.0 to run, 8.0 to stair-climbing, 7.0 to jogging, bicycling, lap swimming, and playing tennis and other racquet sports, 6.0 to aerobics and calisthenics, and 2.5 to 4.5 to walking, depending on the pace were assigned. Then based on the total PA, the quintiles were considered as categories. | Q1 (lowest) = 0.2 to 2.3 METs (h/wk), Q2 = 2.4 to 5.1 METs, Q3 = 5.2 to 11.4 METs, Q4 = 11.5 to 22.8 METs, Q5 (Highest) = 22.9 METs or more. The index of the Highest quintile (>= 22.9) was extracted. | Q1 (lowest) = 0.2 to 2.3 METs (h/wk) | The physical activity questionnaire has been validated in the NHS-II population. | Self-administered questionnaire |
| Lin 2011 | Physical exercise / Vigorous | No Leisure Time Physical Activity (NLTPA) was based on the Physical Activity Scale for the Elderly. The cut-off points to define NLTPA is referred to the American College of Sports Medicine (ACSM) exercise guidelines for older adults, which recommended the older adults perform moderate-intensity physical activity at least 5 times/week. | (1) NLTPA Active (either housework or transportation, at least five times per week) (2) NLTPA Inactive (those who failed to meet this level 5 times p/w). | Active | The Physical Activity Scale for the Elderly - PASE | Face to face home interviews by a well-trained pharmacist using a structured questionnaire |
| Buchman 2012 | Physical exercise / Vigorous | Total daily activity, including both exercise and non-exercise physical activity, was measured at baseline for up to 10 days with actigraphs (Actical, Philips Healthcare) worn on the wrist 24 hours per day. Total daily activity was calculated. Total daily physical activity ranged from 0.06*10^5 counts per day to 13.56*10^5 counts per day with a mean (SD) of 2.88*10^5 (1.57*10^5) counts per day. | In this study the hazard ratio extracted is that of the baseline according to the index of total daily PA. | Total daily PA index = 0 | Measure procedures with actigraph were employed in a previous project | Structured annual clinical examination of the Memory and Aging Project |
| Moore 2012 | Physical exercise / Vigorous | Each of the six cohort studies assessed Leisure-time physical activities (LTPA) through different questionnaires. For all six studies, the energy expended per activity was calculated by multiplying the MET level by the number of hours per week and summed across activities to estimate overall MET hours per week (MET-h/wk) of LTPA energy expenditure. Also, six predefined categories were created to correspond with cut points in the 2008 US federal physical activity guidelines and the 2010 WHO guidelines. | (1) 0 MET-h/wk, (2) 0.1–3.74 MET-h/wk, (3) 3.75–7.4 MET-h/wk, (4) 7.5–14.9 MET-h/wk, (5) 15.0–22.4 MET-h/wk, (6) >= 22.5 MET-h/wk). The index of the highest group (22.5 MET and more) was extracted. | Inactive (0 MET-h/wk) | Three of the six cohort studies used questionnaires adapted from the College Alumni Health Study questionnaire, previously validated. The PA questionnaires in the remaining 3 studies, while not directly validated, but were used in prior studies. | Not mentioned |
| Schultz-Larsen 2012 | Physical exercise / Vigorous | Overall measures of exercise and the number of hours spent per week were assessed: running, swimming, bicycling a stationary bike, aerobic dance, stair steppers, hiking, racket sports, and also periods of rapid walking in daily activities. The PA levels were established on the combination of that items and three categories were settled: (1) Low PA, subjects walked or rode a bicycle less than 30 min p/d and did not participate in exercise (2) Medium PA, subjects either walked or rode a bicycle 31 to 60 min p/day or walked or rode a bicycle 30 min or less p/d at the same time as they spent 60 to 120 min weekly doing exercise (3) High PA, subjects either walked or rode a bicycle more than 60 min p/d or walked or rode a bicycle in average 30 to 60 min at the same time as they spent more than 120 min weekly doing exercise. | (1) Low PA (2) Medium PA (3) High PA. The index of High PA was extracted. | Medium PA | The Brønshøj-Husum Study Protocol | Trained nurses applied standardized instruments during a visit in the participant’s home. |
| Xue 2012 | Physical exercise / Vigorous | 4 exercise activities were involved: walking for exercise, dancing, bowling and exercise (e.g., strengthening activities). Also, 2 lifestyle activities (1) strenuous household (e.g., scrubbing) and (2) outdoor (e.g., gardening, chores) were considered. The level of participation in each activity was assessed by frequency and duration of participation during the past 2 weeks. Then PA was calculated as a continuous variable in kcal/day/kg. The time-based criteria established by the Centre for Disease Control and Prevention and the American College of Sports Medicine (CDC/ACSM) were used to determine the categories: inactive (0 minute/week), moderately active (>0 to <150 minutes/week), and very active (=150 minutes/week). | (1) Inactive (0 minutes per week) (2) Any physical activity (involved by moderately and very active categories). | Any physical activity | Shortened version of the Minnesota Leisure Time Activities Questionnaire | Self-reported questionnaire |
| Bell 2014 | Physical exercise / Vigorous | Physical activity index was calculated through the metabolic work performed in a typical 24-hour day measured at late-life baseline. The PA index cut point was dichotomized at median levels (=30.4) and finally two categories were created. | (1) PA Index >= 30.4 (2) Physical Activity Index > 30.4. | PAI >30.4 | The questionnaire is part of The Honolulu Heart Program protocols | Self-report questionnaire |
| Hamer 2014 | Physical exercise / Vigorous | 3-questions on the frequency of participation in vigorous, moderate, and mild PA. Response options were: more than once per week, once per week, one to three times per month, or hardly ever. Then, participants were shown examples of activities on a card to help them interpret different PA intensities. Examples of mild activities included laundry and home repairs, moderate-intensity activity included gardening, cleaning the car, walking at moderate pace, dancing, and floor or stretching exercises, and vigorous intensity included running/jogging, swimming, cycling, aerobics/gym workout, tennis, and digging with a spade. PA was further categorized into four groups. | PA categories: (1) Inactive (no activity on a weekly basis) (2) Only mild activity at least once a week (3) At least moderate but no vigorous activity at least once a week (4) Any vigorous activity at least once a week. The index of PA vigorous was extracted | Inactive (no activity on a weekly basis) | Physical Activity Questionnaire as part of the English Longitudinal Study of Ageing (ELSA) | Personal interview. |
| Menotti 2014 | Physical exercise / Vigorous | Physical activity was considered only from the occupational working point of view, derived from a simple questionnaire and matching the reported activity with the occupation. Then participants were classified in 3 groups and their energy expenditure related to the occupational working was determined similar to previous study (36): (1) Sedentary people had an energy expenditure of less than 2400 kcal (2) Moderately active people of 2400 to 3000 kcal, and (3) Very active people of over 3000 kcal. | (1) Sedentary (2) Moderate (3) Very active. The index of Sedentary group was extracted. | Very active | Questionnaire used on the census of 1963 by the Italian railroad system | Not mentioned |
| Schnohr 2015 | Physical exercise / Vigorous | Survey questions: weekly quantity of jogging, frequency of jogging, and the subject’s own perception of pace (slow, average, fast). Participants were subdivided into 5 groups according to quantity. | (1) Sedentary non jogger (2) <1 hrs/week (3) 1 to 2.4 hrs/week (<6 METs) (4) 2.5 to 4h hrs/week (6 to <12 METs) (5) >4 hrs/week (>12 METs). The index of the fifth group was extracted. | Sedentary non joggers | The Copenhagen City Heart Study Leisure Time Physical Activity Questionnaire | Self-administered questionnaire. |
| Lee 2016 | Physical exercise / Vigorous | Total daily sitting time was assessed by the next question: During a usual day and night, about how many hours do you spend sitting? Response options were: <4 hours/day, 4 to 5 hours/day, 6 to 7 hours/day, 8 to 9 hours/day, 10 to 11 hours/day, 12 to 13 hours/day, 14 to 15 hours/day, and 16 or more hours/day. To facilitate comparison with similar studies, that scores were initially divided into quartiles of sitting time. | (1) Q1 <= 5 hours/day (2) Q2 = 6 to 9 hours/day (3) Q3 = 10 to 13 hours/day (4) Q4 = 14 or more hours/day. The index of Q4 was extracted. | Q1 <=5 hours/day | Questionnaires used were part of the Women’s Health Initiative (WHI) observational study | Interview or self-reported questionnaire |
| Muller 2016 | Physical exercise / Vigorous | Four questions refer to activity during the past year. (1) Four-point, mutually exclusive, ordered category concerning physical activity at work. (2) The amount of time spent in hours per week for summer and winter separately in each of the following activities: walking, cycling, gardening, do-it-yourself, physical exercise and housework. (3) Whether any of the activities in question 2 were engaged in such that it caused sweating or faster heartbeat and, if so, for how many hours during a typical week. (4) Question about stair climbing. Both occupational and recreational physical activity were combined to calculate the total energy expenditure and to construct a simple Physical Activity Index and categorised it into four groups. | (1) Inactive (2) Moderately inactive (3) Moderately active (4) Active. The index of physically Active was extracted. | Inactive | The short physical activity questionnaire | Not mentioned |
| LaMonte 2018 | Physical exercise / Vigorous | Minutes per day of usual Physical Activity were measured using hip-worn triaxial accelerometers. The data was processed using ActiLife software version 6. The VM (vector magnitude) counts were averaged and reported as mean total PA, an indicator of total PA volume. The VM score was categorized into intensity-specific PA levels. The cut points were derived from the OPAC calibration study. It was required at least 4 days with 10 or more hours per day of awake wear time (convention for compliant wear). | (1) Low light-intensity PA (19 to 225 counts/15 seconds) (2) High light-intensity PA (226 to 518 counts/15 seconds) (3) MVP moderate to vigorous PA (>=519 counts/15 seconds). The index of High PA was extracted. | Low light-intensity PA (19 to 225 counts/15 seconds) | Measure procedures with triaxial accelerometers were used in a previous project | Participants received an accelerometer, wear instructions, and a sleep log. |
| Schnohr 2018 | Physical exercise / Vigorous | Participation and duration per week regarding 8 different types of exercise were included for each of the following sports: tennis, badminton, soccer, jogging, cycling, low-intensity calisthenics (referred to as gymnastics among the Danes), swimming, and health club activities (e.g., treadmill, elliptical trainer, and weights). Then weekly volume was calculated based on the participation and duration min/wk in each sport. Cycling was the most frequent activity within each sport and by far the one with the longest duration followed by the sport itself. In consequence, the index of weekly volume of cycling was extracted. | (1) Sedentary (2) Cycling | Sedentary | The procedures and examinations were determined by CHD epidemiological surveys | Self-administered questionnaire were completed by the participants and checked by the staff. |
| Konlaan 2000 | Leisure Activity | Reading books or periodicals | (1) At least once a week, (2) Occasionally, (3) Rarely. Reading books or periodicals= Rarely | At least once a week | Interview survey of the Project | Participants were interviewed by non-medical interviewers |
| Lan 2006 | Leisure Activity | Exercise condition was measured through question: Did you engage in any kind of leisure activity during the last two weeks? to classify subjects as exercisers (answering YES) or sedentary individuals (answering NO). | ES_1=Exerciser | Sedentary | Reliability and Validity were informed. | All participants were interviewed with standard questionnaires by well-trained interviewers. |
| Lan 2006 | Leisure Activity | Number of leisure physical activity, 13 activities were identified: walking leisurely, jogging or race walking, swimming leisurely, Chinese style exercise, sports with light effort, aerobic dancing, folk dancing, bicycling leisurely, mountain climbing, weight lifting with light effort, walking upstairs, other indoor exercises, miscellaneous exercises. | (1) Sedentary (2) One (3) Two and over. ES_3=Two and over | Sedentary | Reliability and Validity were informed. | All participants were interviewed with standard questionnaires by well-trained interviewers. |
| Lin 2011 | Leisure Activity | Leisure Time Physical Activity (LTPA) was measured through question about the frequency and duration of performing twelve common activities: calisthenics or tai chi, gardening, walking for pleasure, bicycling, jogging, hiking, aerobic dancing, folk dancing, tennis, swimming, golf, and miscellaneous exercise (physical activities developed by Ainsworth et al). For each subject, LTPA energy expenditure in MET-hours per kilogram body weight was estimated by summing up the energy expenditure of all activities on a weekly basis. Then the subjects were divided into two groups. | LTPA nonparticipants (MET hour/ week = 0) and LTPA participants (MET hour/ week > 0). ES_1 = nonparticipants | LTPA participants (MET hour/ week > 0) | Compendium of physical activities by Ainsworth | Face to face home interviews by a well-trained pharmacist using a structured questionnaire |
| Lee 2013 | Leisure Activity | Physical Activity Level (PAL) in the ACL is designed to measure the frequency of engaging in three common types of PA: How often do you typically work in the garden or yard? Response options were based on 4-point Likert scale. | (1) Never (2) Rarely (3) Sometimes (4) Often. The index of Gardening often was extracted | Never gardening | Physical Activity Level questionnaire is part of the Americans’ Changing Lives Longitudinal Study | Self-reported questionnaires |
| Li 2014 | Leisure Activity | LTPA was measured through occupational, household and recreational activity. The intensity of PA was calculated based on the metabolic equivalent (MET) values. A MET was defined as the ratio of work metabolic rate to a standard metabolic rate of (4.184 kJ). For each reported activity, a MET value was assigned according to the Compendium of Leisure time physical activity was measured by a combination of walking, cycling and other sports. Finally, the median of LTPA in MET-hours/week was used to categorize participants. | (1) Low (<36 METS hrs/wk), (2) High (>=36 METS hrs/wk). Leisure physical activity = Low | High (>=36 METS hrs/wk) | The short physical activity questionnaire | Not mentioned |
| Aichele 2016 | Leisure Activity | Questions about amount of time spent in hours per month in 14 different types of leisure activity (e.g., housework, exercise, driving). Participants gave details of how they spend their time during an average day. A factor analysis was conducted to obtain a latent variable which was named “Leisure Activity”. Then, a multilevel growth model was conducted to derive individual scores of baseline performance (intercepts at age 70) and life span change. Leisure Activity intercepts were considered to calculate the standardized effect size estimates as predictor of mortality risk. | In this study the hazard ratio extracted is that of the baseline of Leisure Activities. The value of the intercept (standardized unit) of Leisure activity was -0.22 [-0.41, -0.02]. And the effect size is based on 1 standard deviation above the intercept. | Leisure activity = -0.22 [-0.41, -0.02] | The Personal Details Questionnaire (PDQ) which was part of The University of Manchester longitudinal study | Self-reported questionnaire. |
| Schnohr 2018 | Leisure Activity | The type of PA in leisure time (LTPA). The specific sports studied were tennis, badminton, soccer, jogging, cycling, calisthenics, swimming, and health club activities | (1) Sedentary (2) Health club activities (participation and duration min/wk) | Sedentary | The procedures and examinations were determined by CHD epidemiological surveys | Self-administered questionnaires were completed by the participants and checked by the staff. |
| Larsson 2019 | Leisure Activity | Sedentary leisure-time was measured by the numbers of hours per day of TV viewing and/or sitting reading. Response options were between <1 h/d to >6 p/d. | (1) <1 h/d, (2) 1 to 2 h/d, (3) 3 to 4 h/d, (4) 5 to 6 h/d, (5) >6 h/d. The index of the highest Sedentary leisure-time (>6 h/d) was extracted | <1 h/d of TV viewing and/or sitting reading | The TV viewing or sitting reading question was part of the Self-reported total physical activity | Self-administered questionnaire |
| Mok 2019 | Leisure Activity | Habitual physical activity was assessed with a reference time frame of the past year. First question, occupational physical activity, classified as five categories: unemployed, sedentary (e.g., desk job), standing (e.g., shop assistant, security guard), physical work (e.g., plumber, nurse), and heavy manual work (e.g., construction worker, bricklayer). Second open question (referred to LTPA), the time spent (hours/week) on cycling, recreational activities, sports, or physical exercise, separately for winter and summer. Quasi-continuous and marginalized values of PAEE^1^ (Physical activity energy expenditure) in units of kJ/kg/day were derived. The resulting calibration equation was: PAEE^1^ (kJ/kg/day) = 0 (sedentary or no job) + 5.61 (standing job) + 7.63 (manual job) + 0 (no LTPA) + 3.59 (LTPA of 0.1 to 3.5 hours per week) + 7.17 (LTPA of 3.6 to 7 hours per week) + 11.26 (LTPA >7 hours per week). | In this study the hazard ratio extracted is that of the baseline according to the calibration equation employed for PAEE^1^. | 0 kJ/kg/day | Questions are extracted of the Simple physical activity and the objective measure of PAEE^1^ was similar to The short physical activity questionnaire | Clinic visits and interviews carried out by trained nurses. |
| Harkanen 2020 | Leisure Activity | Leisure time physical activity was assessed through the question How much do you exercise and stress yourself physically in your leisure time? | (1) Inactive (2) Low (3) High.  The index of high LTPA was extracted | Inactive | Based on a survey from the National FINRISK Study criteria | Questionnaire |
| Li 2020 | Leisure Activity | Leisure activities in the past 6 months, including watching TV or listening to the radio, playing cards or mahjong, reading newspapers or books, keeping domestic animals or pets, gardening, and attending religious activities. Answers were coded as (a) Almost every day, (b) Sometimes, or (c) Never. The binary participation in the leisure activities variable was coded as 1 = Almost every day or sometimes and 0 = Never. The index score of six leisure activities, ranged 0 to 6 points, was calculated. | (1) Index score = 0 (2) Index score = 1 (2) Index score = 2 | Index score = 0 | The questionnaire was part of the Chinese Longitudinal Healthy Longevity Survey (CLHLS) | Face-to-face interviews performed by a well-trained enumerator or local doctor/nurse. If respondents were unable to answer questions, the information was collected from proxy respondents (a spouse or close family member). |
| Rakowski 1992 | Physical exercise / Walking | How often do you walk a mile or more at a time, without resting? (Probed to determine per week. Both questions measured the actual pattern of behaviour. | (1) Walking >4 days/wk (2) Walking 2 to 3 days/wk (3) Walking 1 days/wk (4) Walking <1 day/Never. Th index of the last category (<1 day /never) was extracted. | Walking >4 days/wk | Self-assessment of Activity and Exercise. | Self-reported questionnaires |
| Bath 1998 | Physical exercise / Walking | Participants were asked about their total walking per typical day. For the analyses, walking and shopping were combined into a single measure of total walking. Then subjects were divided in two groups. | (1) Walking <10min (2) Walking >=10min. The index of Walking <10 min p/d was extracted | >=10min | Customary physical activity | The detailed activity inventories were administered by trained interviewers. |
| Hakim 1998 | Physical exercise / Walking | Distance walked (miles/day), participants were asked about the average distance they walked per day. The response options were presented in miles, 1 mile per day equivalents 1.6 km. | (1) Distance walked = 0 to 0.9 miles (2) Distance walked = 1 to 2 miles (3) Distance walked = 2.1 to 8 miles. The last index was extracted (2.1 to 8 miles) | Distance walked < 1 mile per day | Questionnaire similar from The Framingham Study | Not mentioned |
| Paffenbarger 1998 | Physical exercise / Walking | Number of kilometres of walking per week | (1) <5 km/wk (2) 5 to 14 km/wk (3) >=15 km/wk. The index fo the highest amount of Walking >=15 km/wk was extracted | <5 km/wk | Physical Activities Questionnaire | Self-reported questionnaires were provided by postal surveys |
| Rockhill 2001 | Physical exercise / Walking | Walking and more vigorous non-walking physical activities. The cumulative average measures of hours per week spent walking and hours spent doing more vigorous activities were created. Participants with both less than 1 hour per week of walking and less than 1 hour per week of more strenuous activities composed the reference group. | PA <1h/w and Walking <1h/w. ES_2 = PA >=3 h/w and Walking >=3h/w. | PA <1h/w and Walking <1h/w | Protocol from The Nurses’ Health Study | Participants were nurses and they returned a mailed questionnaire about their medical histories and lifestyles. |
| Gregg 2003 | Physical exercise / Walking | Number of city-blocks or equivalent (12 blocks = 1 mile [1.6 km]) walked per day for exercise or as part of the normal routine. Walking kcal/week at baseline, categories by quintile: | (1) Q1 <70 Kcal (2) Q2= 70 to 186 Kcal (3) Q3 = 187 to 419 Kcal (4) Q1 = 420 to 897 Kcal (5) Q5 >= 898 Kcal. The index of Q5 was extracted. | Q1 < 70 kcal | A modified version of The Harvard Alumni Questionnaire | Not mentioned |
| Landi 2008 | Physical exercise / Walking | Walking intensity information was obtained using a single question: How many minutes do you walk each day?. Response options were 1 = never or less than 30 min, 2 = 30 min, 3 = 1h, 4 = More than 1h. For analytic purposes, participants were categorized in two groups. | (1) Non-walkers or very light walkers (less than 1h/day, score 1 or 2) and (2) Moderate or intense walkers (1h or more p/day, score 3 or 4). | Non-walkers or very light walkers (less than 1h/day) | Walking single item was part the Invecchiare in Chianti Study | Self-administered questionnaire |
| Sun 2010 | Physical exercise / Walking | Walking score was calculated with a MET value of 2.5 to 4.5, depending on the pace were assigned. Then based on that score, the Walking quintiles were considered as categories. | Q1 = 0 to 0.5 METs, Q2 = 0.6 to 2.5 METs, Q3 = 2.7 to 4.5 METs, Q4 = 5 to 11.2 METs, Q5 >= 12.5 METs. The index of Q5 Walking was extracted. | Q1 Walking (0 to 0.5 METs) | The physical activity questionnaire has been validated in the NHS-II population | Self-administered questionnaire |
| Fortes 2013 | Physical exercise / Walking | Participants were asked to report habitual physical activities of the last year. Sports and other activities were asked as type of activity, hours per week spent on it, and period of the year in which the activity is normally performed. Walking was classified in two categories. | (1) Low walking <4 times per week (2) High walking >=4 times per week. The index of High walking was extracted. | Low <4 times p/wk | Questionnaire was adapted from The physical activity questionnaire for the elderly | Self-administered questionnaire |
| Lee 2013 | Physical exercise / Walking | How often do you walk? Participants were instructed to respond using a 4-point Likert scale. | (1) Never (2) Rarely (3) Sometimes (4) Often. The index of Walking often was extracted | Never walking | Physical Activity Level questionnaire is part of the Americans’ Changing Lives Longitudinal Study | Self-reported questionnaires |
| Bell 2014 | Physical exercise / Walking | Walking was measured through the numbers of blocks walked per day. Then that measure was dichotomized at the median level (12 blocks per day) and finally two categories were created. | (1) Walking <12 blocks per day (2) Walking >=12 blocks per day. | Walking >=12 blocks p/day | The questionnaire is part of The Honolulu Heart Program protocols | Self-report questionnaire |
| Zhao 2015 | Physical exercise / Walking | Walking duration was assessed based on self-reported time spent daily walking by asking “How many hours a day do you walk?”. The available options included<30 minutes/day, 30 minutes to 1 hour/day, 1–2 hours/day and =2 hours/day. Time spent on daily walking was clearly defined in the questionnaire to include walking for exercise, working, and household, social, or other activities. | (1) Daily walking <0.5 h/d (2) Daily walking = 0.5 to 1h/d (3) Daily walking = 1 to 2h/d (4) Daily walking >=2h/d | Daily walking <0.5 h/d | Questionnaire was part of the New Integrated Suburban Seniority Investigation Project | Self-administered questionnaire |
| Chudasama 2019 | Physical exercise / Walking | Objective PA was measured using the Axivity AX3 wrist-worn triaxial accelerometer, where participants were requested to wear the monitor continuously for seven consecutive days. We described PA of each tertile in terms of walking at a brisk pace for exercise. Based on the median (interquartile range) value of the lowest tertile equated to 4.3 (2.9-7.2) mins/day of walking at brisk pace. Similarly, the middle tertile equated to 10.1 (7.2-14.4) mins/day; and upper tertile to 21.6 (15.8-30.2) mins/day of brisk walking. Finally, the categories were established. | (1) low (4 min/day), (2) moderate (10 min/day), and (3) high (22 min/day). ES_3 = High walking | low (4 min/day) | UK Biobank physical activity monitor (accelerometer), version 1. 0, 2016 | Participants were requested to wear the monitor continuously for seven consecutive days. |
| Haveman-Nies 2003 | Healthy Diet | Dietary quality groups were based on the Mediterranean Diet Score. The score included: monounsaturated-to-saturated fat ratio, alcohol, legumes, nuts, or seeds, cereals, vegetables and fruits, meat and meat products, and dairy products. The modified Mediterranean diet score ranged from 0 (low-quality diet) to 7 (high-quality diet). | Two dietary groups were composed: (1) Low-dietary quality (scores = 4 or less) (2) High-dietary quality (scores greater than 4). The index of the high-quality diet was extracted. | Low-dietary quality | Mediterranean Diet Score | Questionnaire |
| Seccareccia 2003 | Healthy Diet | Vegetable consumption information included the type of vegetables eaten each day and the portion size of each serving. Data were categorized into 5 classes, including raw vegetables, cooked vegetables, tomatoes, peppers, and eggplant. Then, all vegetables were pooled together, and the average daily consumption (g/day) was calculated. Legumes were not included in this category. | Vegetable consumption (1 ) 0 to 20 gr/day (2) 21 to 40 gr/day (3) 41 to 60 gr/day (4) more than 60 gr/day. The index of more than 60 gr/day was extracted. | 0 to 20 gr/day | The dietary history method | Dieticians visited participants’ homes to observe eating habits and interview them using a dietary history interview sheet. In addition, a 7-day food-use diary was filled, and it was used to cross-check information obtained in the interviews. |
| Willcox 2004 | Healthy Diet | Energy intake was measured by 24-hour diet recall method. Total Energy Intake per day calories was calculated in quintiles. | (1) Q1 = 512 to 1701 (2) Q2 = 1705 to 2061 (3) Q3 = 2065 to 2366 (4) Q4 = 2368 to 2806 (5) Q5 = 2807 to 6480. The index of the fifth quintile (Q5) was extracted. | Q3 = 2065 to 2366 p/day calories | 24-hour diet recall method | Dietary assessment was performed at baseline by a dietitian. |
| Trichopoulou 2005 | Healthy Diet | Mediterranean diet Score is a scale indicating the degree of adherence to the traditional Mediterranean diet. People whose consumption of vegetables, legumes, fruits, cereals, and fish was below the median consumption were assigned a value of zero, and a value of one otherwise. People whose consumption of meat and dairy products was below the median consumption, and people whose with low ethanol consumption per day (men consuming from 10g to less than 50 g, women consuming from 5g to 25g) was assigned a value of one, and a value of zero otherwise. And lipid intake was calculated through the ratio of the sum of monounsaturates and polyunsaturates to saturates. | Mediterranean diet score: (1) Score 0 to 3 (2) Score 4 to 5 (3) Score 6 to 9. The index of Mediterranean diet score equal to 6 to 9 was extracted. | Score 0 to 3 | Mediterranean Diet Score (Trichopoulou et al) | Questionnaire |
| Khaw 2008 | Healthy Diet | Fruit and vegetable intake was measured by blood vitamin C. The plasma vitamin C level was considered as a good biomarker of plant food intake. A blood value of 50 mmol/l or more indicates an intake of at least five servings of fruit and vegetables daily. | Vitamin C >= 50 mmol/l, coded as 1.    Vitamin C < 50 mmol/l, coded as 0. | Vitamin C < 50 mmol/l | Vitamin C assays was part of the EPIC-Norfolk cohort protocol | Plasma vitamin C was measured from blood drawn into citrate bottles. Plasma vitamin C concentration was estimated using a fluorometric assay within 1 week of sampling. |
| Jankovic 2014 | Healthy Diet | The Healthy Diet indicator (HDI) components were obtained through percentages of energy intake from saturated fatty acids, polyunsaturated fatty acids (PUFAs), mono- and disaccharides, protein, intakes of cholesterol (mg/day), fruits and vegetables combined (g/day), and either total dietary fibre or no starch polysaccharides (g/day). All participants with PUFA intakes above the recommended range received 0 points. For the fruits and vegetables components (>25 g/day of fibre and >400 g/day), participants with lower intakes were allocated proportionately fewer points, with 0 g/day as the minimum. Finally, the HDI score was calculated, the maximum HDI score was 70 points if all guidelines were met, the minimum HDI score was 0. | (1) HDI score with 10 points increase (2) Minimum HDI score (score = 0) | Minimum HDI (score = 0) | Different dietary assessment methods were used in each cohort. Translation of foods into nutrients was performed by using cohort-specific food composition tables. Finally, the Healthy Diet Indicator (HDI) was based on the WHO's 2003 Dietary Guidelines with Huijbregts' procedures | Food frequency questionnaire and validated dietary history method were used. Those are interview-derived or self-reported dietary assessments. |
| Li 2014 | Healthy Diet | Usual consumption levels of 148 food items over the last 12 months before recruitment were collected using the food frequency questionnaire (FFQ). Potentially important food groups were focused on, namely, processed/red meat, vegetables/fruits, cereals, fish, and dairy products. These food groups, except processed/red meat, were dichotomized as high and low by using their medians as the cut-off points. For processed/red meat, the cut-off point was 120 g/day. | Vegetable fruit consumption categories: (1) Low (<200 g/d), (2) High (>=200 g/d). | High (>=200 g/d) | Food frequency questionnaire (FFQ) validated by 24-hr dietary recalls | Subjects who responded initial invitation by mail were by interviewed by telephone and completed the basic examination for the study. |
| Menotti 2014 | Healthy Diet | Eating habits were derived from the dietary history method. The complete questionnaire contains 17 food groups, and they were analysed by factor analysis. | (2) The factor scores of Eating habits were divided into 3 tertiles: (1) Diet score = 1 (Unhealthy diet) (2) Diet score = 2 (3) Diet score = 3 (roughly corresponding to the Mediterranean Diet). The index of Diet score = 1 was extracted. | Diet score = 3 | Eating habits diet Score | Questionnaire administered by 3 experienced dietitians |
| Roswall 2015 | Healthy Diet | Healthy Nordic Food Index (HNFI) contains 6 food groups: wholegrain bread, oatmeal, apples/pears, cabbages, root vegetables, fish/shellfish. They were defined including foods which were part of the Food Frequency Questionnaire (FFQ). The score is from 0 to 6 points. | HNFI score: (1) Lower adherers (0 to 1 point) (2) Middle adherers (2 to 3 points) (3) High adherers (4 to 6 points). The index of high adherers was extracted. | 0 to 1 point (Lowest) | Healthy Nordic Food Index (HNFI) | Self-register questionnaire |
| Shi 2015 | Healthy Diet | A question about food frequency intake (both current and at the age of 60) of fruit, vegetable, meat, fish, beans, tea, garlic, egg, and salt-preserved vegetables. | Vegetable intake (1) Never (2) Occasionally (3) Daily. The estimate of daily vegetables consumption was extracted | Never | A standard questionnaire from the Chinese Longitudinal Healthy Longevity Survey | Self-reported information was collected by face-to-face interviews (at home) by trained research staff. |
| Muller 2016 | Healthy Diet | The dietary score used was an adapted version of the WCRF/AICR^2^ score, including the intakes of (1) energy dense foods/sugary drinks, (2) plant foods (fruits/vegetables/dietary fibre), and (3) animal foods (red and processed meat). These dietary components were graded according to the cut-points. Based on this scoring, participant diets were graded from 0 to 3, from which sex-specific fourths were created. | Dietary score groups: unhealthy, moderately unhealthy, moderately healthy, and healthy. The index of Healthy group was extracted. | Unhealthy | Diet intake over the previous 12 months were assessed using validated country/centre-specific dietary questionnaires | Questionnaire |
| Daskalopoulou 2018 | Healthy Diet | Dietary habits were assessed by asking the number of fruit and vegetable servings in the last 3-days. | Daily consumption level: (1) Consumption of 3 or more servings the last 3 days (2) non-daily consumption, 0 to 2 servings the last 3 days | Non-daily consumption (0 to 2 servings in the last 3-days) | Survey based on the 10/66 Dementia Research Group's protocol | Interviews were generally carried out in participants' own homes, the full assessment, lasting approximately 2–3 hours. |
| Chudasama 2020 | Healthy Diet | A healthy diet was based on eating at least 5 portions of a variety of fruit and vegetables every day. Combined responses were used for fresh fruit (pieces), dried fruit (pieces), salad/raw vegetable (heaped tablespoons), and cooked vegetable (heaped tablespoons). These portions were grouped as 5 portions/day (meet fruit/vegetable guidelines) or <5 portions/day (do not meet fruit/vegetable guidelines) | Yes = Healthy diet, at least 5 portions of fruit and vegetables every day. No = Diet < 5 portions of fruit and vegetables every day | Diet < 5 portions of fruit and vegetables every day | NHS guideline | Questionnaire information through in-person interviews at the UK Biobank Centre. |
| Harkanen 2020 | Healthy Diet | The variables indicating dietary variables covering fresh vegetables and fruits, type of bread spread and type of milk. | Eat fresh vegetables: (1) At most twice a week (2) Almost daily (3) Daily. The estimate of daily vegetables consumption was extracted | At most twice a week | Based on a survey from the National FINRISK Study criteria | Questionnaire |
| Goto 2003 | Sleeping 7hrs | Hours of sleep was part of the health behaviour and social activity questions on the interview. | (1) Less than 6 hours (2) 6 to 7 hours (3) More than 7 hours. The index of first and third categories were extracted | 6 to 7 hours | Health status assessment questionnaire of the elderly, developed by the Tokyo Metropolitan Institute of Gerontology | Personal interviews by epidemiologists, psychologists, physicians, public health workers, and university students from the Tokyo Metropolitan Institute of Gerontology. |
| Yeo 2013 | Sleeping 7hrs | People were asked about their average sleeping time per day. | (1) Less or equal to 5 hours p/day (2) 6 hours p/day (3) 7h hours p/day (4) 8 hours p/day (5) 9h hours p/day (6) More o equal to 10 hours. The index of first and sixth categories were extracted | 7 hours | Structured questionnaire based on the study's protocol of the Korean Multi-centre Cancer Cohort (KMCC) | Direct interview conducted by well-trained personnel. |
| Cao 2020 | Sleeping 7hrs | Information on sleep duration was collected by asking the following question: “How many hours on average do you sleep every day, including napping?” | (1) Short sleep = Less than 7 hours (2) Moderate sleep = 7 to 9 hours (3) Long sleep = More than 9 hours. The index of short and long sleep were extracted | Moderate sleep = 7 to 9 hours | Questionnaire based on the Chinese Longitudinal Healthy Longevity Surveys (CLHLS) protocol. | Direct interview. |

^1^ PAEE = Physical activity energy expenditure (PAEE), ^2^ WCRF/AICR = World Cancer Research Fund/American Institute for Cancer Research guidelines
