# Supplementary material for: Behavioral Lifestyles and Survival: A Meta-Analysis
Source: Front Psychol. 2022 Feb 4;12:786491. doi: 10.3389/fpsyg.2021.786491 (PMC8854179; doi:10.3389/fpsyg.2021.786491)
Supplement: Supplementary file 3 [file Table_3.docx]

# Supplementary Material Table S3. Detailed search strategy - through April 30th, 2021

| **Bibliographic Database** | **Search’s equation** | |
| --- | --- | --- |
| Web of Science (Clarivate) | #1 | (TS = ("Longevity" OR "Life Expectancy")) |
|  | #2 | (TS = (Lifestyle OR “Life style”)) |
|  | #3 | (TS = (“Physical activity” OR “Exercise" OR "Diet, Healthy" OR "Body Weight" OR "Body-Weight Trajectory" OR "Body Weight Changes" OR "Body Weight Maintenance" OR "Sleep Hygiene/classification" OR "Sleep/classification" OR "Sleep/epidemiology" OR "Sleep/psychology" OR "Healthy Lifestyle" OR "Sedentary Behavior") |
|  | #4 | (TS = (“Longitudinal Stud*” OR "Follow-Up Stud*" OR “twin stud*” OR "prospective stud*” OR “meta analy*” OR “Twin stud*”)) |
|  | #5 | (AB = ("Aged")) |
|  | #6 | (SILOID==("WOS")) |
|  | Sequence | **#7: #**2 OR #3  **#8:** #1 AND #7  **#9:** ((#8 AND #4) AND #5)  **#10:** #9 AND #6 |
| MedLine (Clarivate) | #1 | (TS = ("Longevity" OR "Life Expectancy")) |
|  | #2 | (TS = (Lifestyle OR “Life style”)) |
|  | #3 | (TS = (“Physical activity” OR “Exercise" OR "Diet, Healthy" OR "Body Weight" OR "Body-Weight Trajectory" OR "Body Weight Changes" OR "Body Weight Maintenance" OR "Sleep Hygiene/classification" OR "Sleep/classification" OR "Sleep/epidemiology" OR "Sleep/psychology" OR "Healthy Lifestyle" OR "Sedentary Behavior")) |
|  | #4 | (TS = (“Longitudinal Stud*” OR "Follow-Up Stud*" OR “twin stud*” OR "prospective stud*” OR “meta analy*” OR “Twin stud*”)) |
|  | #5 | (AB = ("Aged")) |
|  | #6 | (SILOID == ("MEDLINE")) |
|  | Sequence | **#7: #**2 OR #3  **#8:** #1 AND #7  **#9:** ((#8 AND #4) AND #5)  **#10:** #9 AND #6 |
| Scopus | 1 | TITLE-ABS-KEY ("Longevity" OR "Life Expectancy") |
|  | 2 | TITLE-ABS-KEY (Lifestyle OR “Life style”) |
|  | 3 | TITLE-ABS-KEY (“Exercise" OR "Activities of Daily Living" OR "Functional Status" OR "Diet, Healthy" OR "Body Weight" OR "Body-Weight Trajectory" OR "Body Weight Changes" OR "Body Weight Maintenance" OR "Sleep Hygiene/classification" OR "Sleep/classification" OR "Sleep/epidemiology" OR "Sleep/psychology" OR "Healthy Lifestyle" OR "Sedentary Behavior") |
|  | 4 | TITLE-ABS-KEY (“Longitudinal Stud*” OR "Follow-Up Stud*" OR “twin stud*” OR "prospective stud*” OR “meta analy*” OR “Twin stud*”) |
|  | 5 | ABS("Aged") |
|  | Sequence | **#6: #**2 OR #3  **#7:** ((#1 AND #6**)** AND #5) |
| PubMed | #1 | ("Longevity"[Mesh] OR "Life Expectancy"[Mesh]) |
|  | #2 | ("Life Style"[Mesh]) |
|  | #3 | (Physical activity[Text Word] OR "Exercise"[Mesh] OR "Diet, Healthy"[Mesh] OR "Body Weight"[Mesh] OR "Body-Weight Trajectory"[Mesh] OR "Body Weight Changes"[Mesh] OR "Body Weight Maintenance"[Mesh] OR "Sleep Hygiene/classification"[Mesh] OR "Sleep/classification"[Mesh] OR "Sleep/epidemiology"[Mesh] OR "Sleep/psychology"[Mesh]) |
|  | #4 | ("Healthy Lifestyle"[Mesh] OR "Sedentary Behavior"[Mesh]) |
|  | #5 | ("Longitudinal Stud*"[Mesh] OR "Follow-Up Stud*"[Mesh] OR "prospective stud*"[MeSH Terms] OR “prospective study”[Text Word] OR **"Twin Studies as Topic/methods"[Mesh] OR** "meta-analysis"[Publication Type] OR "meta-analysis as topic"[MeSH Terms]) |
|  | #6 | ("Aged"[Mesh]) |
|  | Sequence | **#7: #**2 OR #3 OR #4  **#8:** #1 AND #7  **#9:** ((#8 AND #5) AND #6) |
| PsycInfo | #1 | (MM ("Longevity" OR "Life Expectancy")) |
|  | #2 | (MM (Lifestyle OR “Life style”)) |
|  | #3 | (MM (“Physical activity” OR “Exercise" OR "Diet, Healthy" OR "Body Weight" OR "Body-Weight Trajectory" OR "Body Weight Changes" OR "Body Weight Maintenance" OR "Sleep Hygiene/classification" OR "Sleep/classification" OR "Sleep/epidemiology" OR "Sleep/psychology" OR "Healthy Lifestyle" OR "Sedentary Behavior")) |
|  | #4 | AG ( aged OR very old ) |
|  | Sequence | **#5: #**2 OR #3  **#6:** ((#1 AND #5) AND #4) |
