# Supplementary material for: Behavioral Lifestyles and Survival: A Meta-Analysis
Source: Front Psychol. 2022 Feb 4;12:786491. doi: 10.3389/fpsyg.2021.786491 (PMC8854179; doi:10.3389/fpsyg.2021.786491)
Supplement: Supplementary file 4 [file Data_Sheet_1.docx]

**Supplementary Material Table S4.** **References**

Studies included in the meta-analysis are denoted in the References with an asterisk.

*Aichele, S., Rabbitt, P., & Ghisletta, P. (2016). Think Fast, Feel Fine, Live Long: A 29-Year Study of Cognition, Health, and Survival in Middle-Aged and Older Adults. *Psychological Science, 27*(4), 518–529. https://doi.org/10.1177/0956797615626906

Bales, C.W., Ritchie, C.S. (2002). Sarcopenia, weight loss, and nutritional frailty in the elderly. *Annu Rev Nutr. 22*,309–323. DOI: 10.1146/annurev.nutr.22.010402.102715

*Bath, P. A., & Morgan, K. (1998). Customary physical activity and physical health outcomes in later life. *Age and Ageing, 27*(SUPPL. 3), 29–34. https://doi.org/10.1093/ageing/27.suppl_3.29

Bjornerem A, Straume B, Midtby M, et al. (2004). Endogenous sex hormones in relation to age, sex, lifestyle factors, and chronic diseases in a general population: the Tromso Study. *J Clin Endocrinol Metab.;89*(12):6039–47. https://doi.org/10.1210/jc.2004-0735.

Blane, D. (1995). Social determinants of health –Socioeconomic status, social class and ethnicity. *American J. Public Health,85*, 903-904. doi: 10.2105/ajph.85.7.903

Blázquez, D., Botella, J., and Suero, M. (2017). The debate on the ego-depletion effect: Evidence from meta-analysis with the p-uniform method. Frontiers in Psychology, 8, 197. DOI: 10.3389/fpsyg.2017.00197

Borenstein, M., Hedges, L. V., Higgins, J., & Rothstein, H. R. (2010). A basic introduction to fixed-effect and random-effects models for meta-analysis. *Research Synthesis Methods, 1*, 97–111. doi: 10.1002/jrsm.12.

*Buchman, A. S., Yu, L., Boyle, P. A., Shah, R. C., & Bennett, D. A. (2012). Total Daily Physical Activity and Longevity in Old Age. *Arch Intern Med., 172*(5), 444–446.

*Byberg, L., Melhus, H., Gedeborg, R., Sundström, J., Ahlbom, A., Zethelius, B., Berglund, L. G., Wolk, A., & Michaëlsson, K. (2009). Total mortality after changes in leisure time physical activity in 50 year old men: 35 Year follow-up of population based cohort. *BMJ (Online), 338*(7700), 936. https://doi.org/10.1136/bmj.b688

Cai, H., Shu, X. O., Xiang, Y. B., Yang, G., Li, H., Ji, B. T., Gao, J., Gao, Y. T., & Zheng, W. (2015). Sleep duration and mortality: a prospective study of 113 138 middle-aged and elderly Chinese men and women. *Sleep, 38*(4), 529–536. https://doi.org/10.5665/sleep.4564

*Cao, Z., Dintica, C., Shang, Y., Cheng, Y., Li, S., Yang, H., Sun, L., Xu, W., & Wang, Y. (2020). The Role of Cognitive Impairment, Physical Disability, and Chronic Conditions in the Association of Sleep Duration with All-Cause Mortality Among Very Old Adults. *Journal of the American Medical Directors Association, 21*(10), 1458-1463.e2. https://doi.org/10.1016/j.jamda.2020.02.017

*Carlsson, A. C., Theobald, H., & Wändell, P. E. (2010). Health factors and longevity in men and women: A 26-year follow-up study. *European Journal of Epidemiology, 25*(8), 547–551. https://doi.org/10.1007/s10654-010-9472-2

Carter, M. I., & Hinton, P. S. (2014). Physical activity and bone health. *Missouri medicine, 111*(1), 59–64.

*Cevenini, E., Cotichini, R., Stazi, M. A., Toccaceli, V., Palmas, M. G., Capri, M., De Rango, F., Dato, S., Passarino, G., Jeune, B., & Franceschi, C. (2014). Health status and 6 years survival of 552 90+ Italian sib-ships recruited within the EU Project GEHA (GEnetics of Healthy Ageing). *Age*, *36*(2), 949–966. https://doi.org/10.1007/s11357-013-9604-1

*Chakravarty, E. F., Hubert, H. B., Krishnan, E., Bruce, B. B., Lingala, V. B., & Fries, J. F. (2012). Lifestyle risk factors predict disability and death in healthy aging adults. *American Journal of Medicine*, *125*(2), 190–197. https://doi.org/10.1016/j.amjmed.2011.08.006

Chaput, J. P., Dutil, C., & Sampasa-Kanyinga, H. (2018). Sleeping hours: what is the ideal number and how does age impact this?. *Nature and science of sleep, 10*, 421–430. https://doi.org/10.2147/NSS.S163071

Cherkas, L.F., Hunkin, J.L., Kato, B.S., et al. (2008). The association between physical activity in leisure time and leukocyte telomere length. *Archives of Internal Medicine.168*(2), 154–8

Christensen, K., and Vaupel, J.W. (1996) Determinants of longevity: genetic, environmental and medical factor. *Journal of Internal Medicine*, *240*: 333–341

Christensen, K., Doblhammer, G., Rau, R., and Vaupel, J.W. (2009) . Ageing populations: the challenges ahead.  *Lancet*, *374*: 1196–208374.

*Chudasama, Y. V., Khunti, K. K., Zaccardi, F., Rowlands, A. V., Yates, T., Gillies, C. L., Davies, M. J., & Dhalwani, N. N. (2019). Physical activity, multimorbidity, and life expectancy: A UK Biobank longitudinal study. *BMC Medicine*, *17*(1), 1–13. https://doi.org/10.1186/s12916-019-1339-0

*Chudasama, Y. V., Khunti, K., Gillies, C. L., Dhalwani, N. N., Davies, M. J., Yates, T., & Zaccardi, F. (2020). Healthy lifestyle and life expectancy in people with multimorbidity in the UK Biobank: A longitudinal cohort study. *PLoS Medicine*, *17*(9), 1–18. https://doi.org/10.1371/journal.pmed.1003332

*Chyou, P. H., Burchfiel, C. M., Yano, K., Sharp, D. S., Rodriguez, B. L., Curb, J. D., & Nomura, A. M. Y. (1997). Obesity, alcohol consumption, smoking and mortality. *Annals of Epidemiology*, *7*(4), 311–317. https://doi.org/10.1016/S1047-2797(97)00019-7

Colpani, V., Baena, C.P., Jaspers, L., van Dijk, G.M., Farajzadegan, Z., Dhana, K., Tielemans, M.J., Voortman, T., Freak-Poli, R., Veloso, G.G.V., Chowdhury, R., Kavousi, M., Muka, T., and Franco, O.H.(2018). Lifestyle factors, cardiovascular disease and all-cause mortality in middle-aged and elderly women: a systematic review and meta-analysis. European J. Epidemiology, 33: 831-845. <https://doi.org/10.1007/s10654-018-0374-z>.

*Daskalopoulou, C., Koukounari, A., Ayuso-Mateos, J. L., Prince, M., & Prina, A. M. (2018). Associations of lifestyle behaviour and healthy ageing in five latin American and the Caribbean countries—A 10/66 population-based cohort study. *Nutrients*, *10*(11). https://doi.org/10.3390/nu10111593

*Dhana, K., Berghout, M. A., Peeters, A., Ikram, M. A., Tiemeier, H., Hofman, A., Nusselder, W., Kavousi, M., & Franco, O. H. (2016). Obesity in older adults and life expectancy with and without cardiovascular disease. *International Journal of Obesity*, *40*(10), 1535–1540. https://doi.org/10.1038/ijo.2016.94

Di Angelantonio E, Bhupathiraju ShN, Wormser D, Gao P, Kaptoge S, Berrington de Gonzalez A, Cairns BJ, Huxley R, Jackson ChL, Joshy G, Lewington S, Manson JE, Murphy N, Patel AV, Samet JM, Woodward M, Zheng W, Zhou M, Bansal N, Barricarte A, Carter B, Cerhan JR, Smith GD, Fang X, Franco OH, Green J, Halsey J, Hildebrand JS, Jung KJ, Korda RJ, McLerran DF, Moore SC, O'Keeffe LM, Paige E, Ramond A, Reeves GK, Rolland B, Sacerdote C, Sattar N, Sofianopoulou E, Stevens J, Thun M, Ueshima H, Yang L, Yun YD, Willeit P, Banks E, Beral V, Chen Zh, Gapstur SM, Gunter MJ, Hartge P, Jee SH, Lam TH, Peto R, Potter JD, Willett WC, Thompson SG, Danesh J, Hu FB. (2016). Body-mass index and all-cause mortality: individual-participant-data meta-analysis of 239 prospective studies in four continents. *Lancet.;388*(10046):776-86. doi: 10.1016/S0140-6736(16)30175-1.

Dowd, J.B., Goldman, N,. and Weinstein, M. (2011). Sleep duration, sleep quality, and biomarkers of inflammation in a Taiwanese population. *Ann Epidemiol;21*(11):799-806. doi: 10.1016/j.annepidem.2011.07.004.

*Dutta, A., Henley, W., Lang, I., Llewellyn, D., Guralnik, J., Wallace, R. B., & Melzer, D. (2011). Predictors of extraordinary survival in the Iowa established populations for epidemiologic study of the elderly: Cohort follow-up to “ extinction.” *Journal of the American Geriatrics Society*, *59*(6), 963–971. https://doi.org/10.1111/j.1532-5415.2011.03451.x

*Edjolo, A., Helmer, C., Barberger-Gateau, P., Dartigues, J. F., Maubaret, C., & Peres, K. (2013). Becoming a nonagenarian: Factors associated with survival up to 90 years old in 70+ men and women. Results from the paquid longitudinal cohort. *Journal of Nutrition, Health and Aging*, *17*(10), 881–892. https://doi.org/10.1007/s12603-013-0041-8

Ennour-Idrissi, K., Maunsell, E., and Diorio, C. (2015). Effect of physical activity on sex hormones in women: a systematic review and meta-analysis of randomized controlled trials. *BCR.;17*(1):139. <https://doi.org/10.1186/s13058-015-0647-3>.

*Evans, M. F., & Frank, J. (1997). Body weight and mortality among women. *Canadian Family Physician Médecin de Famille Canadien*, *43*(11), 455. https://doi.org/10.1097/00006205-199511000-00011

*Fang, J., Wylie-Rosett, J., & Alderman, M. H. (2005). Exercise and cardiovascular outcomes by hypertensive status: NHANES I epidemiological follow-up study, 1971-1992. *American Journal of Hypertension*, *18*(6), 751–758. https://doi.org/10.1016/j.amjhyper.2004.12.020

*Feng, Q., Hoenig, H. M., Gu, D., Yi, Z., & Purser, J. L. (2010). Effect of new disability subtype on 3-year mortality in Chinese older adults. *Journal of the American Geriatrics Society*, *58*(10), 1952–1958. https://doi.org/10.1111/j.1532-5415.2010.03013.x

Fernández-Ballesteros, R. and Botella, J. (2007). Self-report measures. In Nezu & Nezu (Eds.). *EvidenceBased Outcome Research: A Practical Guide to Conducting Randomized Controlled Trials*. Oxford University Press.

Fernández-Ballesteros, R. (2017). “Psicología y Envejecimiento: El comportamiento humano, ¿un factor causal de longevidad?,” in Academia de Psicologia de España. Psicología para un mundo sostenible: Volumen I (Madrid: Pirámide), 81–116

Fernández-Ballesteros, R. and Sánchez-Izquierdo, M. (2019). Are Psycho-Behavioral Factors Accounting for Longevity?. *Frontiers in Psychology 10*, 2516. doi: 10.3389/fpsyg.2019.02516

Fernandez-Ballesteros, R. (2019) *Psycho-Behavioural factors: The missing link for a new paradigm to account for active Longevity (PsyBeL*). National Agency of Research.

*Ford, J., Spallek, M., & Dobson, A. (2008). Self-rated health and a healthy lifestyle are the most important predictors of survival in elderly women. *Age and Ageing*, *37*(2), 194–200. https://doi.org/10.1093/ageing/afm171

*Fortes, C., Mastroeni, S., Sperati, A., Pacifici, R., Zuccaro, P. G., Francesco, F., Agabiti, N., Piras, G., Amleto, D., & Ebrahim, S. (2013). Walking four times weekly for at least 15 min is associated with longevity in a Cohort of very elderly people. *Maturitas*, *74*(3), 246–251. https://doi.org/10.1016/j.maturitas.2012.12.001

*Franco, O. H., de Laet, C., Peeters, A., Jonker, J., Mackenbach, J., & Nusselder, W. (2005). Effects of Physical Activity on Life Expectancy With Cardiovascular Disease. *Archives of Internal Medicine*, *165*(20), 2355–2360. https://doi.org/10.1016/j.amjcard.2011.08.042

*Fries, J. F. (1998). Effects of Regular Walking on Mortality among Nonsmoking Retired Men. *Clinical Journal of Sport Medicine*, *8*(3), 249. https://doi.org/10.1097/00042752-199807000-00022

Fries, J.F., and Crapo, (1981) Prevention Policy for the Elderly Care. *Scandinavian Journal of Primary Health Care*, 3:1,pp 8-9, DOI: 10.3109/02813438509017729.

*Godshalk, A. N., & Brown, M. M. (2007). Midlife risk factors and healthy survival in men - Commentary. *Evidence-Based Ophthalmology*, *8*(2), 90–91. https://doi.org/10.1097/IEB.0b013e31803c9aae

*Goto, A., Yasumura, S., Nishise, Y., & Sakihara, S. (2003). Association of health behavior and social role with total mortality among Japanese elders in Okinawa, Japan. *Aging Clinical and Experimental Research*, *15*(6), 443–450. https://doi.org/10.1007/BF03327366

*Grand, A., Grosclaude, P., Bocquet, H., Pous, J., & Albaredez, J. L. (1990). Disability, psychosocial factors and mortality among the elderly in a rural french population. *Journal of Clinical Epidemiology*, *43*(8), 773–782. https://doi.org/10.1016/0895-4356(90)90237-J

Greenberg, J.A., Fontaine, K., and Allison, D.B. (2007) Putative biases in estimating mortality attributable to obesity in the US population. *Int J Obes (Lond).;31*(9):1449–1455.

*Gregg, E. W., Cauley, J. A., Stone, K., Thompson, T. J., Bauer, D. C., Cummings, S. R., & Ensrud, K. E. (2003). Relationship of Changes in Physical Activity and Mortality among Older Women. *Journal of the American Medical Association*, *289*(18), 2379–2386. https://doi.org/10.1001/jama.289.18.2379

Gremeaux, V., Gayda, M., Lepers, R., Sosner, P., Juneau, M., and Nigam, A. (2012). Exercise and longevity. *Maturitas,73*, 312–7. doi: 10.1016/j.maturitas.2012.09.012.

*Gulsvik, A. K., Thelle, D. S., Mowé, M., & Wyller, T. B. (2009). Increased mortality in the slim elderly: A 42 years follow-up study in a general population. *European Journal of Epidemiology*, *24*(11), 683–690. https://doi.org/10.1007/s10654-009-9390-3

*Gulsvik, A. K., Thelle, D. S., Samuelsen, S. O., Myrstad, M., Mowé, M., & Wyller, T. B. (2012). Ageing, physical activity and mortality-a 42-year follow-up study. *International Journal of Epidemiology*, *41*(2), 521–530. https://doi.org/10.1093/ije/dyr205

Hamer, M., and Chida, Y. (2008). Walking and primary prevention: a metaanalysis of prospective cohort studies. *Br J Sports Med. 42*(4), 238–43. <https://doi.org/10.1136/bjsm.2007.039974>

*Hamer, M., De Oliveira, C., & Demakakos, P. (2014). Non-exercise physical activity and survival: English Longitudinal Study of Ageing. *American Journal of Preventive Medicine*, *47*(4), 452–460. https://doi.org/10.1016/j.amepre.2014.05.044

Hankin, J. (2000). Lifestyles and health. In E.Borgatta and R. Montgomery (eds.). *Encyclopedia of Sociology* (pp. 1639-1643). New York: Macmillan.

*Härkänen, T., Kuulasmaa, K., Sares-Jäske, L., Jousilahti, P., Peltonen, M., Borodulin, K., Knekt, P., & Koskinen, S. (2020). Estimating expected life-years and risk factor associations with mortality in Finland: Cohort study. *BMJ Open*, *10*(3), 1–10. https://doi.org/10.1136/bmjopen-2019-033741

*Haveman-Nies, A., de Groot, L. C. P. G. M., & van Staveren, W. A. (2003). Dietary quality, lifestyle factors and healthy ageing in Europe: The SENECA study. *Age and Ageing*, *32*(4), 427–434. https://doi.org/10.1093/ageing/32.4.427

*Heir, T., Erikssen, J., & Sandvik, L. (2013). Life style and longevity among initially healthy middle-aged men: Prospective cohort study. *BMC Public Health*, *13*(1). https://doi.org/10.1186/1471-2458-13-831

Hendriks J., and Hatch LR. (2006). Lifestyles and Aging. In Binstock, R.H. and George, L.K. Eds.) *Handbook of Aging and the Social Sciences,* pp 301-319. SanDiego CA. Academic Press.

Housman, J., and Dorman, S. (2005). The Alameda County Study: A Systematic, Chronological Review. *American Journal of Health Education, 36*(5), 302-308.

Hu, F.B., Stampfer, M.J., Colditz, G.A., and Ascherio, A. (2000). Physical activity and risk of stroke in women. *JAMA.;283*(22), 2961–7. https://doi.org/10.1001/jama.283.22.2961.

Huedo-Medina, T. B., Sánchez-Meca, J., Marín-Martínez, F. y Botella, J. (2006). Assessing heterogeneity in meta-analysis: Q statistic or I2 index? Psychological Methods, 11(2), 193-206. doi: 10.1037/1082-989X.11.2.193.

*Jankovic, N., Geelen, A., Streppel, M. T., De Groot, L. C. P. G. M., Orfanos, P., Van Den Hooven, E. H., Pikhart, H., Boffetta, P., Trichopoulou, A., Bobak, M., Bueno-De-Mesquita, H. B., Kee, F., Franco, O. H., Park, Y., Hallmans, G., Tjønneland, A., May, A. M., Pajak, A., Malyutina, S., … Feskens, E. J. (2014). Adherence to a healthy diet according to the world health organization guidelines and all-cause mortality in elderly adults from Europe and the United States. *American Journal of Epidemiology*, *180*(10), 978–988. https://doi.org/10.1093/aje/kwu229

*Keadle, S. K., Arem, H., Moore, S. C., Sampson, J. N., & Matthews, C. E. (2015). Impact of changes in television viewing time and physical activity on longevity: A prospective cohort study. *International Journal of Behavioral Nutrition and Physical Activity*, *12*(1), 1–11. https://doi.org/10.1186/s12966-015-0315-0

Keith, S.W., Fontaine, KR, Allison, DB (2013). Mortality rate and overweight: Overblown or underestimated? A commentary on a recent meta-analysis of the associations of BMI and mortality. *MOLECULAR METABOLISM, 2*(2), 65-68. DOI10.1016/j.molmet.2013.03.005

*Khaw KT, Wareham N, Bingham S, Welch A, Luben R, et al. (2008) Combined Impact of Health Behaviours and Mortality in Men and Women: The EPIC-Norfolk Prospective Population Study. PLOS Medicine 5(3): e70. <https://doi.org/10.1371/journal.pmed.0050070>

*Kinge, J. M., & Morris, S. (2014). Variation in the relationship between BMI and survival by socioeconomic status in Great Britain. Economics and Human Biology, 12(1), 67–82. https://doi.org/10.1016/j.ehb.2013.05.006

*Konlaan, B. B., Bygren, L. O., & Johansson, S. E. (2000). Visiting the cinema, concerts, museums or art exhibitions as determinant of survival: A Swedish fourteen-year cohort follow-up. Scandinavian Journal of Public Health, 28(3), 174–178. https://doi.org/10.1177/14034948000280030501

La Vecchia, C., and Tavani, A. (1998). Fruit and vegetables, and human cancer. *Eur J Cancer Prev.7*, 3–8.

*LaMonte, M. J., Buchner, D. M., Rillamas-Sun, E., Di, C., Evenson, K. R., Bellettiere, J., Lewis, C. E., Lee, I. M., Tinker, L. F., Seguin, R., Zaslovsky, O., Eaton, C. B., Stefanick, M. L., & LaCroix, A. Z. (2018). Accelerometer-Measured Physical Activity and Mortality in Women Aged 63 to 99. *Journal of the American Geriatrics Society*, *66*(5), 886–894. https://doi.org/10.1111/jgs.15201

*Lan, T. Y., Chang, H. Y., & Tai, T. Y. (2006). Relationship between components of leisure physical activity and mortality in Taiwanese older adults. *Preventive Medicine*, *43*(1), 36–41. https://doi.org/10.1016/j.ypmed.2006.03.016
